# Clinical practice guideline on pregnancy and renal disease

**DOI:** 10.1186/s12882-019-1560-2

**Published:** 2019-10-31

**Authors:** Kate Wiles, Lucy Chappell, Katherine Clark, Louise Elman, Matt Hall, Liz Lightstone, Germin Mohamed, Durba Mukherjee, Catherine Nelson-Piercy, Philip Webster, Rebecca Whybrow, Kate Bramham

**Affiliations:** 1 NIHR Doctoral Research Fellow in Obstetric Nephrology, Guy’s and St. Thomas’ NHS Foundation Trust and King’s College London, London, UK; 2 Guy’s and St. Thomas’ NHS Foundation Trust and King’s College London, London, UK; 3 King’s College Hospital NHS Foundation Trust, London, UK; 4 Expert Patient, c/o The Renal Association, Bristol, UK; 5 Nottingham University Hospital, Nottingham, UK; 6 Imperial College London and Imperial College Healthcare NHS Trust, London, UK; 7 Guy’s and St. Thomas’ NHS Foundation Trust and Imperial College Healthcare NHS Trust, London, UK; 8 Imperial College Healthcare NHS Trust, London, UK; 9 King’s College London, London, UK; 10 King’s College Hospital NHS Foundation Trust and King’s College London, London, UK

## Introduction

### Background

Chronic kidney disease (CKD) is estimated to affect 3% of pregnant women in high-income countries, (Piccoli et al., 2018, #13860) which equates to between 15,000–20,000 pregnancies per year in England. The prevalence of CKD in pregnancy is predicted to rise in the future due to increasing maternal age and obesity.

Although CKD is not a barrier to reproduction in most women, the risk of adverse pregnancy outcomes is increased in women with CKD including pre-eclampsia, fetal growth restriction, preterm delivery and accelerated loss of maternal renal function. CKD impacts on communication, decision-making, and the surveillance and management of women before, during, and after pregnancy.

Existing guidance on the management of CKD in pregnancy includes the UK Consensus Group on Pregnancy in Renal Disease (ISBN 978–1,107,124,073) and expert review. Neither Kidney Disease Outcomes Quality Initiative (KDOQI) or National Institute of Health and Care Excellence (NICE) have produced specific guidance on the management of renal disease in pregnancy. Published guidance containing information relevant to the care of women with CKD in pregnancy includes:

- KDIGO 2017 Clinical Practice Guideline Update for the Diagnosis, Evaluation, Prevention, and Treatment of Chronic Kidney Disease–Mineral and Bone Disorder (CKD-MBD). UK Renal Association Commentary available at: BMC Nephrology 2018; 19: 240.
- KDOQI Clinical Practice Guideline for Haemodialysis, 2015.
- KDIGO Clinical Practice Guideline for the Evaluation and Management of CKD, 2012
- KDIGO Clinical Practice Guideline for Glomerulonephritis, 2012
- KDIGO Guideline for the Care of Kidney Transplant Recipients, 2009.
- KDIGO Clinical Practice Guidelines for Nutrition in Chronic Renal Failure, 2008.
- NICE: Intrapartum Care for Women with Existing Medical Conditions or Obstetric Complications and their Babies [NG121], 2019.
- NICE: Urinary Tract Infection (Lower) Antimicrobial Prescribing [NG109], 2018
- NICE: Urinary Tract Infection (Recurrent) Antimicrobial Prescribing [NG112], 2018.
- NICE: Antenatal Care for Uncomplicated Pregnancies [CG62], 2008, updated 2017.
- NICE: Vitamin D supplement use in specific population groups [PH56], 2017
- NICE: Diabetes in Pregnancy: Management from Pre-conception to the Post-partum Period [NG3], 2015.
- NICE: Antenatal and postnatal mental health: clinical management and service guidance [CG192], 2014, updated 2018.
- NICE: Fertility: Assessment and Treatment for People with Fertility Problems, 2013.
- NICE: Weight management before, during and after pregnancy [PH27], 2010 [additional data from 2017 surveillance available at: https://www.nice.org.uk/guidance/ph11/evidence/appendix-a-summary-of-evidence-from-surveillance-pdf-4671107966]
- NICE: Hypertension in Pregnancy: Diagnosis and Management [CG107], 2011 (update awaited 2019).
- UK Renal Association Clinical Practice Guidelines: Undernutrition in Chronic Kidney Disease, June 2019
- RCOG: Thrombosis and Embolism During Pregnancy and the Puerperium, Reducing the Risk [Green-Top Guideline 37a], 2015.
- MBBRACE Confidential Enquiry into Maternal Deaths and Morbidity: lessons learned to inform maternity care (triennial reports)
- www.european-renal-best-practice.org/content/erbp-documents

### Aims

The aim of this guideline is to improve the standard of, and to reduce regional variation in, the care of women with CKD in the UK who are pregnant, planning a pregnancy or post-partum.

### Scope

This guidance covers the care of women with CKD (including renal transplant recipients) who are planning a pregnancy, pregnant, or in the post-partum period. It also covers contraception and fertility for women with CKD.

This guideline can be used in the following settings:

- General practice
- Community and hospital antenatal clinics
- Antenatal, labour and postnatal wards
- Renal out-patients
- Renal wards
- Dialysis units

The target audience and intended users of this guideline are nephrologists, obstetricians, obstetric physicians, midwives, renal nurses, pharmacists, specialist trainees in both nephrology and obstetrics, and women with CKD who are pregnant or considering pregnancy. Qualitative data on the experience of pregnancy and renal disease is provided in Appendix 1. A summary of clinical responsibility for elements of the guideline is provided in Appendix 2.

The clinical issues covered in this guideline are:

### Structure of care

#### Medication

#### Pre-pregnancy care

3.1. Contraception
3.2. Fertility
3.3. Pre-pregnancy counselling and optimisation for pregnancy

#### Pregnancy care

4.1 Assessment of renal function in pregnancy
4.2 Antenatal care
4.3 Pre-eclampsia prophylaxis
4.4 Blood pressure management
4.5 Thromboembolism prophylaxis
4.6 Anaemia
4.7 Bone health
4.8 Renal biopsy
4.9 Peripartum care
4.10 Postnatal care

#### Specific conditions

5.1 Renal transplantation
5.2 Dialysis
5.3 Lupus
5.4 Diabetic nephropathy
5.5 Urinary tract infection (UTI)
5.6 Congenital Abnormalities of the Kidney and Urinary Tract (CAKUT)

Clinical issues that will not be covered are acute kidney injury and renal stone disease. In addition, fertility, contraception, teratogenicity and genetic implications in men with CKD will not be addressed.

### Search strategy

Literature searches were undertaken using Ovid Medline (1946 to 2018) using specific search terms related to each of the clinical issues covered in the guideline. Search terms are detailed in Appendix 3.

## Summary of clinical practice guidelines

### Structure of care

#### Guideline 1.1

We recommend multidisciplinary teams (including a consultant obstetrician, consultant nephrologist/expert physician, and expert midwife or midwifery team) are established to offer advice and care for women with CKD who are pregnant or planning a pregnancy. All healthcare professionals caring for women with CKD should be able to access this MDT (1D).

### Medication in pregnancy and lactation

#### Guideline 2.1

We recommend that low dose aspirin, low molecular weight heparin, labetalol, nifedipine, methyldopa, prednisolone, azathioprine, ciclosporin, tacrolimus and hydroxychloroquine are safe for use in pregnancy (1B).

#### Guideline 2.2

We recommend concentrations of calcineurin inhibitors (tacrolimus, ciclosporin) are checked throughout pregnancy and immediately postpartum, as blood concentrations may change (1C).

#### Guideline 2.3

We recommend that medications which interfere with calcineurin inhibitor metabolism (e.g. erythromycin, clarithromycin) are avoided in pregnant and post-partum women taking tacrolimus or ciclosporin whenever possible (1D).

#### Guideline 2.4

We recommend mycophenolate mofetil, methotrexate and cyclophosphamide are not taken in pregnancy as they are teratogenic (1B).

#### Guideline 2.5

We recommend mycophenolate mofetil is stopped before pregnancy, as use in pregnancy is associated with an increased risk of spontaneous miscarriage and fetal abnormality. A 3-month interval is advised before conception to allow conversion to a pregnancy-safe alternative and ensure stable disease/kidney function (1C).

#### Guideline 2.6

We recommend that, when other treatment options exist, rituximab is avoided in pregnancy due to the risk of neonatal B cell depletion and unknown long-term outcomes (1D).

#### Guideline 2.7

We recommend sirolimus and everolimus are avoided in pregnancy due to insufficient safety data (1D).

#### Guideline 2.8

We suggest the benefits of eculizumab in pregnancy for organ threatening disease are likely to outweigh risk (2D).

#### Guideline 2.9

We recommend metformin can be used in pregnancy for women with a pre-pregnancy eGFR>30mls/min/1.73m^2^ and stable renal function during pregnancy (1D).

#### Guideline 2.10

We recommend immunosuppressive treatment is not increased routinely in the peripartum period and that dose changes are based on clinical indications and blood concentrations (1D).

#### Guideline 2.11

We recommend women can breastfeed whilst taking prednisolone, hydroxychloroquine, azathioprine, ciclosporin, tacrolimus, enalapril, captopril, amlodipine, nifedipine, labetalol, atenolol and low molecular weight heparin (1C).

### Pre-pregnancy care

#### Contraception

##### Guideline 3.1.1

We recommend advice on safe and effective contraception is offered to all women of reproductive age with CKD (1D).

##### Guideline 3.1.2

We recommend safe and effective contraception is offered to women of reproductive age who are taking teratogenic medication, have active glomerulonephritis, are within one year of renal transplantation or acute graft rejection, and for any woman who does not wish to conceive (1D).

##### Guideline 3.1.3

We recommend that the progesterone only-pill, a progesterone subdermal implant, and the progesterone intra-uterine system are safe and effective for women with CKD (1C).

##### Guideline 3.1.4

We recommend that progesterone-only emergency contraception is safe for women with CKD (1C).

#### Fertility

##### Guideline 3.2.1

We suggest fertility preservation is considered for women of reproductive age who require treatment with cyclophosphamide (2C).

##### Guideline 3.2.2

We suggest women who have had previous treatment with cyclophosphamide have early investigation of infertility (2D).

##### Guideline 3.2.3

We recommend women with CKD are referred for pre-pregnancy counselling before receiving assisted reproduction (1D).

##### Guideline 3.2.4

We recommend single-embryo transfer is performed to reduce risk of complications associated with multifetal pregnancies in women with CKD (1C).

#### Pre-pregnancy counseling and optimization for pregnancy

##### Guideline 3.3.1

We suggest women with CKD considering pregnancy are offered pre-pregnancy counselling by a multidisciplinary team including a consultant obstetrician and nephrologist or expert physician (2D).

##### Guideline 3.3.2

We recommend women with CKD are advised there is an increased risk of complications in pregnancy including pre-eclampsia, preterm birth, fetal growth restriction, and neonatal unit (NNU) admission, and that they are more likely to require caesarean delivery (1C).

##### Guideline 3.3.3

We recommend women with known or suspected inheritable renal diseases are offered genetic counselling including inheritance risk, prognosis, and intervention options including pre-implantation genetic diagnosis (1C).

##### Guideline 3.3.4

We recommend pre-pregnancy counselling for the optimisation of maternal and neonatal outcomes in women with CKD, which may include:

- stabilising disease activity in advance of pregnancy on minimised doses of pregnancy-appropriate medications (1B).
- optimising blood pressure control (< 140/90 mmHg) on pregnancy-appropriate medications (1B).
- optimising glycaemic control in women with diabetes mellitus (1A) (see section 5.4).
- minimising risk of exposure to teratogenic medications (1C) (see section 2).
- making a treatment plan in the event of hyperemesis or disease exacerbation/relapse during pregnancy (1D).

##### Guideline 3.3.5

We recommend women with CKD who are taking angiotensin converting enzyme inhibitors have a plan for discontinuation/conversion guided by the strength of indication for renin-angiotensin blockade and the likelihood of pregnancy confirmation in the first trimester (1B).

##### Guideline 3.3.6

We recommend angiotensin receptor antagonists are discontinued in advance of pregnancy (1D).

##### Guideline 3.3.7

We suggest women with CKD stages 4 and 5 contemplating pregnancy are offered pre-dialysis education (2D).

### Pregnancy Care

#### Assessment of renal function in pregnancy

##### Guideline 4.1.1

We recommend renal function in pregnancy is assessed using serum creatinine concentrations as estimated GFR (eGFR) is not valid for use in pregnancy (1C).

##### Guideline 4.1.2

We recommend women with CKD have formal quantification of proteinuria in pregnancy (1D).

##### Guideline 4.1.3

We recommend quantification of proteinuria is undertaken by protein:creatinine ratio (uPCR) or albumin:creatinine ratio (uACR). Twenty-four hour urine collection for quantification of protein is not required (1B).

#### Antenatal care

##### Guideline 4.2.1

We suggest pregnant women with CKD who have not had pre-pregnancy counselling by the MDT are referred to the MDT and receive the same counselling and optimisation as for women attending pre-pregnancy (2D).

##### Guideline 4.2.2

We recommend pregnant women with CKD receive routine antenatal care, in addition to specialist input (1D).

##### Guideline 4.2.3

We recommend pregnant women with CKD be referred for assessment by a consultant obstetrician (1D).

##### Guideline 4.2.4

We recommend pregnant women with CKD have access to usual trisomy screening with specialist interpretation of high-risk results (1C).

##### Guideline 4.2.5

We recommend women with CKD exposed to teratogenic drugs in the first trimester are referred to a specialist fetal medicine unit (1D).

##### Guideline 4.1.6

We recommend pregnant women with CKD have scans to assess fetal growth and wellbeing in the third trimester (1C).

##### Guideline 4.2.7

We recommend pregnant women taking prednisolone and/or calcineurin inhibitors are screened for gestational diabetes (1C).

#### Pre-eclampsia prophylaxis

##### Guideline 4.3.1

We recommend women with CKD are offered low-dose aspirin (75-150 mg) in pregnancy to reduce the risk of pre-eclampsia (1B).

##### Guideline 4.3.2

We suggest kidney donors are offered low dose aspirin (75 mg–150 mg) to reduce the risk of pre-eclampsia (2D).

#### Blood pressure management

##### Guideline 4.4.1

We recommend that the target blood pressure during pregnancy for women with CKD is 135/85 mmHg or less, which should be documented in the woman’s healthcare record (1D).

##### Guideline 4.4.2

We suggest antihypertensive treatment in women with CKD is continued in pregnancy unless systolic blood pressure is consistently < 110 mmHg systolic, or diastolic blood is pressure consistently < 70 mmHg diastolic BP, or there is symptomatic hypotension (2D).

##### Guideline 4.4.3

We recommend labetalol, nifedipine and methyldopa can be used to treat hypertension in pregnancy (1B).

##### Guideline 4.4.4

We recommend angiotensin converting enzyme inhibitors, angiotensin receptor antagonists and diuretics are not used to treat hypertension in pregnancy (1B).

##### Guideline 4.4.5

We recommend a diagnosis of superimposed pre-eclampsia is considered:

- in a woman with non-proteinuric CKD, if she develops new hypertension (systolic BP > 140 mmHg and/or diastolic BP > 90 mmHg) and proteinuria (uPCR > 30 mg/mmol or uACR > 8 mg/mmol) or maternal organ dysfunction after 20 weeks’ gestation (1B).
- in a women with proteinuric CKD if she develops new hypertension (systolic BP > 140 mmHg and/or diastolic BP > 90 mmHg) or maternal organ dysfunction after 20 weeks’ gestation (1B)
- in a women with chronic hypertension and proteinuria, if she develops maternal organ dysfunction after 20 weeks’ gestation (1B).

##### Guideline 4.4.6

We suggest in women with chronic hypertension and proteinuria that the development of sustained severe hypertension (systolic BP > 160 mmHg and/or diastolic BP > 110 mmHg or doubling of antihypertensive agents) and/or a substantial rise in proteinuria (doubling of uPCR or uACR compared to early pregnancy) should prompt clinical assessment for superimposed pre-eclampsia (2D).

##### Guideline 4.4.7

We suggest a role for angiogenic markers (PlGF±sFlt-1) is considered as an adjunct to diagnose superimposed pre-eclampsia, dependent upon on-going research in women with CKD (2C).

#### Venous thromboembolism

##### Guideline 4.5.1

We recommend that women with nephrotic-range proteinuria (uPCR> 300 mg/mmol or ACR > 250 mg/mmol) be offered thromboprophylaxis with low molecular weight heparin in pregnancy and the post-partum period unless there is a specific contraindication including risk of labour or active bleeding (1D).

##### Guideline 4.5.2

We suggest that non-nephrotic range proteinuria in pregnancy is a risk factor for thrombosis and thromboprophylaxis with low molecular weight heparin should be considered in the presence of additional risk factors (2D).

#### Anaemia

##### Guideline 4.6.1

We recommend pregnant women with CKD are given parenteral iron if indicated (1C).

##### Guideline 4.6.2

We recommend erythropoietin stimulating agents are given if indicated in pregnancy (1C).

#### Bone health

##### Guideline 4.7.1

We recommend women with CKD who are vitamin D deficient be given vitamin D supplementation in pregnancy (1B).

##### Guideline 4.7.2

We recommend calcimimetics are discontinued in pregnancy (1D).

##### Guideline 4.7.3

We recommend non-calcium based phosphate binders are discontinued in pregnancy (1D).

#### Renal biopsy

##### Guideline 4.8.1

We recommend if a histological diagnosis will change management in pregnancy then renal biopsy can be performed in the first and early second trimester of pregnancy (1C).

#### Peripartum care

##### Guideline 4.9.1

We recommend women with CKD receive routine peripartum care, with additional specialist input (1D).

##### Guideline 4.9.2

We recommend women with CKD have observations taken and documented during any hospital admission. This includes temperature, heart rate, blood pressure, respiratory rate, and oxygen saturation. An early warning score should be calculated and actioned appropriately (1D).

##### Guideline 4.9.3

We recommend additional assessment for women with an elevated early warning score, for women considered to be high-risk, and for any women in whom there is any clinical concern. This includes examination of jugular venous pressure, lung auscultation and urine output monitoring (in-dwelling catheter not usually required) in addition to routine parameters (1D).

##### Guideline 4.9.4

We recommend women with CKD at risk of volume depletion or volume overload are highlighted by the MDT in advance of delivery (1D).

##### Guideline 4.9.5

We recommend that fluid balance is managed with the aim of maintaining normal fluid volume, avoiding dehydration and pulmonary oedema, with input from clinicians with expertise in fluid balance and renal disease (1D).

##### Guideline 4.9.6

We recommend all clinicians are aware of the increased risk of pulmonary oedema in women with CKD and pre-eclampsia (1D).

##### Guideline 4.9.7

We recommend the timing of birth for women with CKD is determined by obstetric indications, with consideration of renal factors including deteriorating renal function, symptomatic hypoalbuminaemia, pulmonary oedema, and refractory hypertension (1D).

#### Postnatal care

##### Guideline 4.10.1

We recommend that non-steroidal anti-inflammatories should not be given (1C).

##### Guideline 4.10.2

We recommend women with CKD have a planned early postpartum renal review (1D).

##### Guideline 4.10.3

We recommend that women with CKD are prescribed medications that are compatible with breastfeeding whenever possible (1D).

##### Guideline 4.10.4

We recommend that women with CKD are offered safe and effective contraception post-partum and receive updated pre-pregnancy counselling before future pregnancies (1D).

### Specific conditions

#### Renal transplantation

##### Guideline 5.1.1

We recommend women with renal transplants wait until their kidney function is stable on medications that are safe in pregnancy before conceiving, which is usually more than one year after transplantation (1D).

##### Guideline 5.1.2

We recommend that plans for delivery in a woman with a renal transplant are discussed with the local surgical transplant team (1D).

##### Guideline 5.1.3

We recommend that mode of delivery in women with renal transplants is based on obstetric indications and maternal preference (1D).

##### Guideline 5.1.4

We recommend that caesarean delivery in a woman with a renal transplant patient is performed by the most senior obstetrician available, ideally a consultant (1D).

##### Guideline 5.1.5

We recommend that women with kidney-pancreas transplants, kidney-liver transplants, and dual kidney transplants are managed during pregnancy and delivery by a multidisciplinary team including transplant physicians and surgeons, at a transplant centre (1D).

#### Dialysis

##### Women receiving maintenance dialysis before pregnancy

**Guideline 5.2.1**

We recommend women established on dialysis prior to pregnancy receive pre-pregnancy counselling including the options of postponing pregnancy until transplantation (when feasible) and the need for long frequent dialysis prior to and during pregnancy (1C).

**Guideline 5.2.2**

We recommend women established on haemodialysis prior to pregnancy receive long, frequent haemodialysis either in-centre or at home to improve pregnancy outcomes (1C).

**Guideline 5.2.3**

We suggest women receiving haemodialysis during pregnancy have dialysis dose prescribed accounting for residual renal function, aiming for a pre-dialysis urea < 12.5 mmol/l (2C).

**Guideline 5.2.4**

We recommend women established on peritoneal dialysis prior to pregnancy should convert to haemodialysis during pregnancy (1D).

##### Initiating dialysis during pregnancy

**Guideline 5.2.5**

We suggest haemodialysis should be initiated in pregnancy when the maternal urea concentration is 17-20 mmol/L and the risks of preterm delivery outweigh those of dialysis initiation. Gestation, renal function trajectory, fluid balance, biochemical parameters, blood pressure and uraemic symptoms should be considered in addition to maternal urea concentration (2D).

#### Lupus nephritis and vasculitis

##### Guideline 5.3.1

We recommend that women with lupus or vasculitis should be advised to wait until their disease is quiescent for at least 6 months before conceiving (1B).

##### Guideline 5.3.2

We recommend that all women with lupus should be advised to take hydroxychloroquine in pregnancy unless it is contraindicated (1C).

##### Guideline 5.3.3

We recommend that women with lupus be monitored for disease activity during pregnancy (1D).

##### Guideline 5.3.4

We recommend that women who are positive for anti-Ro (SSA) or anti-La (SSB) antibodies be referred for fetal echocardiography in the second trimester (1C).

##### Guideline 5.3.5

We recommend women with antiphospholipid syndrome and a history of a confirmed thromboembolic event or previous adverse obstetric outcome (excluding recurrent early fetal loss) receive low molecular weight heparin in pregnancy and for six weeks postpartum (1B).

##### Guideline 5.3.6

We recommend that steroids, azathioprine, calcineurin inhibitors, intravenous immunoglobulin and plasma exchange can be used to treat lupus in pregnancy (1C).

#### Diabetic nephropathy

##### Guideline 5.4.1

We recommend that women with diabetic nephropathy have optimisation of blood glucose, blood pressure and proteinuria prior to conception (1C).

##### Guideline 5.4.2

We recommend that women with diabetic nephropathy continue angiotensin converting enzyme inhibitors until conception, with regular pregnancy testing during attempts to conceive (1C).

##### Guideline 5.4.3

We recommend that the schedule of care, surveillance and management of women with diabetic nephropathy should be untaken according to national guidelines for diabetes in pregnancy, in addition to specialist monitoring of renal disease in pregnancy (1D).

#### Urinary Tract Infection (UTI)

##### Guideline 5.5.1

We suggest women with reflux nephropathy, congenital anomalies of the kidneys and urinary tract (CAKUT), women with CKD taking immunosuppression, and women with a history of recurrent UTI should be offered antibiotic prophylaxis during pregnancy after a single UTI in pregnancy, including asymptomatic bacteriuria (2D).

##### Guideline 5.5.2

We recommend pre-pregnancy UTI prophylaxis be continued in pregnancy using agents known to be safe (1D).

#### Reflux nephropathy and Congenital Abnormalities of the Kidney and Urinary Tract (CAKUT)

##### Guideline 5.6.1

We recommend women with previous bladder surgery (re-implantation of ureter, bladder reconstruction, all complex paediatric urology) should be discussed during pregnancy with a urologist with expertise in bladder reconstruction to evaluate options for delivery (1D).

##### Guideline 5.6.2

We recommend that antenatally detected abnormalities in the fetal kidneys and/or urinary tract should be discussed with fetal medicine and paediatric nephrology specialists to determine appropriate neonatal management (1D).

##### Guideline 5.6.3

We recommend that children with antenatally detected abnormalities in the fetal kidneys and/or urinary tract should have specialist follow up if features of urinary tract infection are identified (1C).

## Summary of audit measures

- **Audit Measure 1:** Proportion of UK renal units and obstetric centres with access to a multidisciplinary team (including a consultant obstetrician, consultant nephrologist/expert physician, and expert midwife or midwifery team) to advise and/or manage renal disease in pregnancy.
- **Audit Measure 2:** Incidence of pregnancies (including spontaneous miscarriage and elective terminations of pregnancy) exposed to mycophenolate mofetil and cyclophosphamide within 6 weeks prior to date of conception.
- **Audit Measure 3:** Proportion of women of reproductive age with CKD within the first year of transplantation offered safe and effective contraception.
- **Audit Measure 4:** Proportion of women of reproductive age with CKD within 6 months of a lupus flare offered safe and effective contraception.
- **Audit Measure 5:** Proportion of women of reproductive age with CKD taking teratogenic medication (mycophenolate mofetil, cyclophosphamide, methotrexate) offered safe and effective contraception.
- **Audit Measure 6:** Proportion of pregnant women with CKD with quantification of proteinuria before 20 weeks’ gestation.
- **Audit Measure 7:** Proportion of women with CKD taking prednisolone or calcineurin inhibitors who are screened for gestational diabetes.
- **Audit Measure 8:** Proportion of pregnant women with CKD offered low dose aspirin (75-150 mg) before 16 weeks’ gestation.
- **Audit Measure 9:** Proportion of pregnant women with CKD that have a target blood pressure for pregnancy documented in their antenatal record.
- **Audit Measure 10:** Proportion of women with CKD given non-steroidal anti-inflammatory drugs in the post-partum period.
- **Audit Measure 11:** Proportion on women with CKD who are breastfeeding their infants (exclusively or mixed feeding) at 6 weeks post-partum.

## Summary of research recommendations

The UK Kidney Research Consortium has highlighted the need for better evidence in order to define care pathways, assess acceptability to patients and ensure optimum outcomes. These recommendations for research are very relevant to pregnancy in women with CKD. The guideline committee therefore suggest the following research recommendations:

**Research recommendation 1:** Qualitative evaluation of methods used to communicate risk in pregnancy in order to achieve understanding of risk and facilitate shared decision-making regarding reproductive health.

**Research recommendation 2:** Establishment of multicentre registries with standardisation of data collection from pregnant women with CKD to allow prospective, large cohort studies to evaluate:

- renal disease aetiology and mechanisms of disease progression
- factors influencing outcomes in women with dialysis, transplantation
- optimal schedule of care in pregnancy
- optimal management of hypertension, proteinuria, medications, anaemia, vitamin D concentrations
- impact of kidney donation on pregnancy and renal outcomes

**Research recommendation 3:** Assessment of the impact of CKD on aneuploidy screening methods including cell-free fetal DNA.

**Research recommendation 4:** Validation of angiogenic (PlGF) and antiangiogenic (sFlt-1) biomarkers in the diagnosis of superimposed pre-eclampsia and prediction of adverse pregnancy outcomes in women with CKD.

**Research recommendation 5:** Evaluation of short- and long-term outcomes in the children of women with CKD, including the excretion into breast milk of medications used in women with CKD.

## Rationale for Clinical Practice Guidelines

### Structure of care

#### Guideline 1.1

We recommend multidisciplinary teams (including a consultant obstetrician, consultant nephrologist/expert physician, and expert midwife or midwifery team) are established to offer advice and care for women with CKD who are pregnant or planning a pregnancy. All healthcare professionals caring for women with CKD should be able to access this MDT (1D).

##### Rationale

Women with CKD have an increased risk of adverse pregnancy outcomes including pre-eclampsia, fetal growth restriction, preterm delivery and deterioration in maternal renal function. A recommendation for expert multidisciplinary care in pregnancy exists for women with other medical comorbidities associated with increased risk in pregnancy including cardiac disease [1], diabetes [2], epilepsy [3], and cancer [4]. It is unlikely that there will ever be randomised trial evidence supporting multidisciplinary care in pregnancy for women with CKD given the lack of perceived equipoise, but it was the consensus opinion of the guideline committee that multidisciplinary team working is critical for optimum care and timely clinical decision-making for women with CKD in pregnancy. The deficiencies in management identified in the care of women with pre-existing medical conditions that die during or shortly after pregnancy have been linked consistently to an absence of coordinated, expert, multidisciplinary care [5, 6]. A MDT is therefore recommended to facilitate informed decision-making regarding pregnancy, and to prevent and/or manage obstetric, renal and neonatal complications that may develop. The MDT should be available prior to, during, and following pregnancy. Options for accessing the MDT include remote advice, face-to-face counselling, and the direct delivery of maternity care.

### Medication in pregnancy and lactation

#### Guideline 2.1

We recommend that low dose aspirin, low molecular weight heparin, labetalol, nifedipine, methyldopa, prednisolone, azathioprine, ciclosporin, tacrolimus and hydroxychloroquine are safe for use in pregnancy (1B).

#### Guideline 2.2

We recommend concentrations of calcineurin inhibitors (tacrolimus, ciclosporin) are checked throughout pregnancy and immediately postpartum, as blood concentrations may change (1C).

#### Guideline 2.3

We recommend that medications which interfere with calcineurin inhibitor metabolism (e.g. erythromycin, clarithromycin) are avoided in pregnant and post-partum women taking tacrolimus or ciclosporin whenever possible (1D).

#### Guideline 2.4

We recommend mycophenolate mofetil, methotrexate and cyclophosphamide are not taken in pregnancy as they are teratogenic (1B).

#### Guideline 2.5

We recommend mycophenolate mofetil is stopped before pregnancy, as use in pregnancy is associated with an increased risk of spontaneous miscarriage and fetal abnormality. A 3-month interval is advised before conception to allow conversion to a pregnancy-safe alternative and ensure stable disease/kidney function (1C).

#### Guideline 2.6

We recommend that, when other treatment options exist, rituximab is avoided in pregnancy due to the risk of neonatal B cell depletion and unknown long-term outcomes (1D).

#### Guideline 2.7

We recommend sirolimus and everolimus are avoided in pregnancy due to insufficient safety data (1D).

#### Guideline 2.8

We suggest the benefits of eculizumab in pregnancy for organ threatening disease are likely to outweigh risk (2D).

#### Guideline 2.9

We recommend metformin can be used in pregnancy for women with a pre-pregnancy eGFR>30mls/min/1.73m^2^ and stable renal function during pregnancy (1D).

#### Guideline 2.10

We recommend immunosuppressive treatment is not increased routinely in the peripartum period and that dose changes are based on clinical indications and blood concentrations (1D).

#### Guideline 2.11

We recommend women can breastfeed whilst taking prednisolone, hydroxychloroquine, azathioprine, ciclosporin, tacrolimus, enalapril, captopril, amlodipine, nifedipine, labetalol, atenolol and low molecular weight heparin (1C).

##### Rationale

Prescribing any medication in pregnancy should involve balancing the risks to the women of uncontrolled disease, with any real or theoretical perceived harm to the fetus. Inappropriate cessation or failure to initiate therapy when clearly indicated can be more harmful than judicious use to maintain maternal health. Medication should be prescribed in pregnancy if the benefit to the woman (and therefore the fetus) outweighs the potential or theoretical risk to the fetus. The woman should be involved in discussions about medication in pregnancy, which should ideally take place before the pregnancy as part of pre–pregnancy counseling [7].

Very few drugs are licensed for use in pregnancy. Surveillance of pregnancy outcomes in women exposed to drugs is therefore used to assess safety in pregnancy. Such outcomes may be confounded by the underlying medical conditions for which treatment is required, and clinical interpretation of data must be balanced and pragmatic. There are no randomised controlled trials of medication in pregnancy in women with CKD. Where randomised controlled trial data are available, they are generalised from unselected or control obstetric cohorts [8, 9].

Table 1 provides a summary of the relevant safety data for medications commonly used in women with CKD in relation to conception, pregnancy and lactation.

**Table 1:** Medication in women with CKD in relation to conception, pregnancy and lactation. Adapted from Wiles et al. [(268]^)^

Drug	Conception (see Section 3.3)	Pregnancy			Lactation	References
		Overall	Maternal considerations	Fetal considerations		
Antihypertensive drugs (see section 4.4)						
Labetalol	Safe	Safe	License for pregnancy. Avoid if asthmatic.	No association with congenital abnormalities. Reduced birth weight in unadjusted observational data. Neonatal bradycardia (2%) and hypoglycaemia (5%).	Safe	[11–16]
Nifedipine	Safe	Safe	None	No association with congenital abnormalities.	Safe	[14, 15]
Amlodipine	Safe	Limited data.	None	Limited data. No adverse effects reported.	Safe	[17, 18]
Methyldopa	Safe	Safe	Avoid in depression or if risk of depression.	No association with congenital abnormalities.	Avoid in all due to risk of postnatal depression.	[14, 15]
Doxazosin	Safe	Limited data	None	No evidence of harm in animal studies	< 1% maternal dose detected.	[15]
Hydralazine	Safe	Safe	Risk of hypotension, tachycardia	No association with congenital abnormalities	Safe	[15]
Beta-blockers	Safe	Limited data on individual drugs	Avoid if asthmatic. Use in pregnancy determined by maternal indication.	No association with congenital abnormalities. Reduced birth weight, clinical significance unclear. Neonatal bradycardia (1%) and hypoglycaemia (3%).	No adverse effects reported	[15, 16, 19–22]
Angiotensin converting enzyme inhibitors	No apparent increase in risk with first trimester use when data are corrected for underlying hypertension. Continue until conception if required for nephroprotection.	Unsafe	None	Fetotoxic in second and third trimesters leading to fetal and neonatal renal failure, bone and aortic arch malformations, oligohydramnios, and pulmonary hypoplasia.	Safety data available for captopril and enalapril.	[14, 15, 23–25]
Angiotensin receptor antagonists	Insufficient data on exposure in early pregnancy. Discontinue in advance of pregnancy.	Unsafe	None	Fetotoxicity in second and third trimesters comparable to angiotensin converting enzyme inhibitors.	No data.	[14, 15]
Thiazide diuretics	Insufficient data on exposure in early pregnancy. No evidence of harm.	Unsafe	Reduced plasma volume expansion in pregnancy (*n* = 10)	No evidence of thrombocytopenia, jaundice, hypokalaemia or hyponatraemia in meta-analysis (*n* = 5292) but advised to avoid.	Potential suppression of lactation. Avoid.	[15, 26, 27]
Immunosuppressant drugs (see Section 5.1 and 5.3)						
Corticosteroids	Safe	Safe	Potential risks: diabetes, hypertension, pre-eclampsia, infection, preterm rupture of membranes. Aim for minimum maintenance dose.	Fetus exposed to < 10% maternal dose due to placental deactivation. No evidence of increase in congenital abnormalities.	Safe. Small amounts in breast milk. Consider timing feeds to 4 h post administration if high dose given (e.g. methylprednisolone induction) and monitor neonate.	[15, 28–31]
Hydroxychloroquine	Safe	Safe	Withdrawal may precipitate lupus flare. Indicated throughout pregnancy if patient has a history of lupus nephritis.	Placental transfer. No increase in miscarriage or congenital abnormality. May reduce risk of congenital heart block if maternal anti-SSA and or anti-SSB antibodies.	Safe	[15, 32–37]
Azathioprine	Safe	Safe	Recommend check TPMT status before dosing.	Placental transfer. No association with congenital abnormalities.	Safe. Low concentration in breast milk	[7, 15, 38–40]
Ciclosporin	Safe	Safe	Monitor pre-dose levels more frequently in pregnancy and immediately post partum. May need higher dose in pregnancy. Avoid medications which interfere with calcineurin inhibitor metabolism (e.g. erythromycin, clarithromycin). Increased risk of gestational diabetes	Placental transfer. No association with congenital abnormalities.	Safe	[15, 41, 42]
Tacrolimus	Safe	Safe	Monitor pre-dose levels more frequently in pregnancy and immediately post-partum. May need a higher dose in pregnancy. Avoid medications which interfere with calcineurin inhibitor metabolism (e.g. erythromycin, clarithromycin). Increased risk of gestational diabetes	Placental transfer. No association with congenital abnormalities.	Safe	[15, 43–45]
Mycophenolate mofetil	Unsafe. Effective contraception during treatment and for 6 weeks after treatment. Ensure disease/transplant stability prior to conception.	Unsafe	None	Placental transfer. Teratogenic causing ear, heart, eye, lip/palate, kidney, and bone abnormalities, tracheoesophageal fistula, congenital diaphragmatic hernia. Increased miscarriage.	Avoid use during lactation due to insufficient data.	[7, 15, 39, 46]
Cyclophosphamide	Unsafe. Effective contraception during and for 3 months after treatment. Dose- and age-related risk of infertility.	Unsafe	None	Placental transfer. Teratogenic. Congenital abnormalities of the skull, ear, face, limb and visceral organs. Increased risk of miscarriage.	Excreted in breast milk. Discontinue breast-feeding during and for 36 h after treatment.	[7, 15, 34, 47, 48]
Rituximab	Unclear (limited data available). Treatment decision depends on indication and alternative options.	Unclear (limited data available)	If indicated for severe disease, aim to give dose before, or in early, pregnancy to minimise the risk of neonatal B-cell depletion.	Active placental transfer in 2nd and 3rd trimester. Potential risk of neonatal B-cell depletion. Avoid unless potential benefit to woman outweighs risk. Long term effects unknown.	Unclear (limited data available). Possible excretion of trace amounts but neonatal absorption unlikely.	[7, 15, 39, 49, 50]
Sirolimus / Everolimus	Unsafe – fetal toxicity in rats. Effective contraception during and for 3 months after treatment.	Unsafe	Impaired wound healing. Proteinuria.	Likely placental transfer. Toxicity in animal studies	Limited data available Avoid.	[15, 51, 52]
Eculizumab	Unclear (limited data available). Treatment decision depends on indication and alternative options.	Unclear (limited data available)	Morbidity of underlying condition may mean treatment in pregnancy is required. Monitor for increased dosage requirements.	Active placental transfer in 2nd and 3rd trimester. No congenital abnormality reported in 20 infants. Long-term effects unknown.	Limited data available. Possible excretion of trace amounts but neonatal absorption unlikely.	[15, 53, 54]
Other drugs						
Aspirin (75–150 mg) (see Section 4.3)	Safe	Safe	Decreases risk of pre-eclampsia in general obstetric population. No evidence of maternal haemorrhagic complications. Insufficient data on optimum dose (i.e. 75 mg versus 150 mg).	No association with congenital abnormalities.	Safe	[8, 9, 14, 15]
Iron (see Section 4.6)	Safe	Safe	Intravenous preparations may offer better bioavailability in CKD	Safety data available in 2nd and 3rd trimesters but limited data on exposure on the first trimester. Expert consensus is not to withhold IV iron if indicated in the first trimester.	Safe	[15, 55–59]
Low-molecular- weight heparin	Safe	Safe	Level of proteinuria which confers a significant risk of VTE in pregnancy is unclear. All pregnant women should be risk assessed for VTE.	No placental transfer.	Safe	[10, 15]
Erythropoietin (see section 4.6)	Safe	Safe	Monitor blood pressure.	No placental transfer.	Safe	[15, 60–62]
Metformin (see Section 5.4)	Safe	Safe	Use contraindicated outside of pregnancy if eGFR < 30 ml/min/1.73m^2^ (approximates to serum creatinine > 150 μmol/L in pregnancy).	None	Levels in milk are low, infants receive < 0.5% of maternal weight-adjusted dosage. No reported adverse effects.	[2, 15]

### Pre-pregnancy care

#### Contraception

##### Guideline 3.1.1

We recommend advice on safe and effective contraception is offered to all women of reproductive age with CKD (1D).

**Rationale**

Although CKD impacts on mechanistic and psychological aspects of fertility, reducing the likelihood of spontaneous conceptions (see section 3.2), unintended pregnancies occur. Although there are no recent data, a historical questionnaire study of 76 women with CKD revealed that despite 50% being sexually active, only 36% used contraception, and only 13% had discussed reproductive health issues with their nephrologist [63]. A survey of 212 women with lupus revealed that 46% were at risk of unintended pregnancy, with 23% having unprotected sex ‘most of the time’. [64] Based on the use of folic acid supplementation at the time of conception, a nationwide survey in the UK estimates that one third of pregnancies in renal transplant recipients are unplanned [65]. Contraceptive counselling of women on dialysis is largely neglected in published literature despite increasing pregnancy rates in contemporary dialysis cohorts, and an association between intensive dialysis and an increased rate of conception [66]. A systematic review of observational studies shows that unintended pregnancy is associated with an increased risk of obstetric complications, even in the absence of co-morbidity [67], with important additional considerations in women with CKD including optimisation of disease management prior to pregnancy, avoidance of teratogenic medication, and providing an awareness of an increased risk of adverse pregnancies outcomes (see Section 3.3).

##### Guideline 3.1.2

We recommend safe and effective contraception is offered to women of reproductive age who are taking teratogenic medication, have active glomerulonephritis, are within one year of renal transplantation or acute graft rejection, and for any woman who does not wish to conceive (1D).

**Rationale**

Exposure to teratogenic medications such as mycophenolate mofetil and cyclophosphamide in the first trimester of pregnancy can lead to abnormalities in the developing fetus (see Section 2). Meta-analyses of observational studies show that active lupus nephritis is a significant risk factor for the development of maternal hypertension and preterm delivery (see Section 5.3) [68, 69]. The first year after transplantation carries the highest risk of rejection, is most likely to require management with teratogenic medication and is associated with adverse pregnancy outcomes (see Section 5.1) [70–72]. All such women should therefore be offered safe and effective contraception.

##### Guideline 3.1.3

We recommend that the progesterone only-pill, a progesterone subdermal implant, and the progesterone intra-uterine system are safe and effective for women with CKD (1C).

##### Guideline 3.1.4

We recommend that progesterone-only emergency contraception is safe for women with CKD (1C).

**Rationale**

The risks and acceptability of different contraceptive methods should be balanced against the risks of an unplanned pregnancy. All oestrogen-containing contraceptives confer risk of hypertension, venous thromboembolism (VTE), arterial thrombosis and cervical cancer [73]. These risks are particularly relevant for women with CKD with co-existing chronic hypertension and those known to be at increased risk of vascular disease, venous thromboembolism (due to anti-phospholipid antibodies or nephrotic syndrome), or cervical neoplasia in the context of immunosuppression. Oestrogen-containing methods are therefore likely to be contraindicated for many women with CKD, particularly given the availability of safer, effective methods.

Progesterone-only methods including the progesterone-only pill (‘mini-pill), the progesterone-containing intrauterine system (Mirena®) and the progesterone subdermal implant (Nexplanon®), do not confer these risks and are therefore considered safe [74]. The ability of the progesterone-only pill to inhibit ovulation varies but one study showed that desogestrel provides consistent inhibition of ovulation in 102 out of 103 women and that this inhibition is maintained even after 12-hour delays before re-dosing [75]. This therapy can therefore be hypothesised to confer improved ‘typical-use’ efficacy over other oral progesterone preparations that require re-dosing within a 3-h window each day.

There is theoretical concern that the efficacy of intrauterine devices is reduced in women taking immunosuppression due to inhibition of uterine inflammation, which is believed to contribute to the underlying contraceptive mechanism. However, the uterine milieu is predominantly populated by macrophages, and immunosuppression used in the management of immune-mediated renal disease and transplantation acts predominantly via lymphocyte inhibition. There is no evidence of an excess of intrauterine device failures following transplantation [76, 77]. Concern regarding pelvic infection in the context of immunosuppression also seems to be unfounded. Data from women with HIV-mediated immunosuppression show no correlation between infective complications and the level of immune suppression measured by CD4^+^ T cell count [78]. A retrospective study of 11 women with renal transplants and a total of 484 months of progesterone-intrauterine device use reported no cases of pelvic infection or unplanned pregnancy [79].

Data on the risk of breast cancer with progesterone methods of contraception are conflicting with a large population study suggesting [80] and a large case-control study refuting [81] a link. Non-hormonal methods (i.e. copper intrauterine device) should be used in women with either a diagnosis or history of breast cancer, and the potential risk of progesterone should be considered in women who are known to have a genetic mutation that confers an increased future risk of breast-cancer [74]. The excess number of cases of breast cases linked to hormone-containing contraceptives is age-related and hormone use should therefore be carefully weighed in women over 40 years [82].

Assessment of contraceptive efficacy should be based on ‘typical use’ rather than presuming ‘perfect use’, as discrepancies exist in the failure rate of some contraceptive methods [83]. Typical use failure rates for the contraceptive pill, implant and progesterone-containing intra-uterine device (Mirena®) are 9, 0.2 and 0.05% respectively within the first year of use. Although barrier methods are effective in preventing transmission of HIV and sexually transmitted disease, 18–21% of couples will conceive within the first year of typical use meaning that condoms cannot be considered to be a reliable, long-term form of contraception for most couples.

In the UK, emergency contraceptive pills (levonorgestrel, ulipristal) do not contain oestrogen and can be safely prescribed in women with CKD within 72 h of unprotected sexual intercourse to prevent pregnancy.

#### Fertility

##### Guideline 3.2.1

We suggest fertility preservation is considered for women of reproductive age who require treatment with cyclophosphamide (2C).

##### Guideline 3.2.2

We recommend women who have had previous treatment with cyclophosphamide have early investigation of infertility (1D).

**Rationale**

Cohort studies show that cyclophosphamide causes age and dose-dependent gonadotoxicity in women with systemic lupus erythematosus [47, 84] and a diminished ovarian reserve (quantified by longitudinal serum AMH concentrations) in women with granulomatosis with polyangiitis [85]. Systematic review data show that, in addition to fertility effects, chemotherapy-induced premature ovarian insufficiency in young women treated for breast cancer has a negative effect on quality of life and is associated with vasomotor symptoms and sexual dysfunction [86]. Fertility preservation should therefore be considered for women of childbearing age receiving cyclophosphamide.

Fertility preservation techniques will depend upon the urgency of treatment of the underlying condition and availability. Cryopreservation of oocytes and gametes can be undertaken, but this usually requires ovarian stimulation, which will typically delay cyclophosphamide administration and, given the immunomodulatory role of oestrogen believed to underlie the female predominance of lupus, carries a theoretical risk of lupus flare. Published data on the risks of ovarian stimulation are limited, conflicting, and there is an absence of prospective trials [87, 88]. Natural cycle in-vitro fertilization (IVF) negates the need for ovarian stimulation and has been described in six patients with nephritis [89]. However, pregnancy rates with natural cycle IVF are lower compared to stimulated cycles and natural cycle retrieval is not recommended for women without CKD [90].

Luteinising hormone releasing hormone analogues (LHRHa)/gonadotrophin releasing hormone agonists (GnRHa) can be used to inhibit the hypothalamic-pituitary-ovarian axis, leading to a protective reduction in ovarian blood flow for the duration of cyclophosphamide treatment. Data on the use of LHRH/GnRHa in women with CKD are limited. A retrospective cohort of 20 women receiving cyclophosphamide (cumulative mean dose 12.5 g) for lupus nephritis showed a reduction in the incidence of premature ovarian failure (amenorrhoea > 12 months and follicle-stimulating hormone level > 40mIU/ml) with use of an LHRH analogue compared to that of age and dose-matched controls (5% versus 30%, respectively) [91]. Most data come from populations treated with chemotherapy for breast cancer with randomised controlled trials [92, 93] and a large meta-analysis of > 1200 patients [94] suggesting that LHRH analogues are safe and effective in reducing premature ovarian failure associated with chemotherapy. In contrast, a recent randomized-controlled trial in young women with lymphoma (mean age 26 years) showed no significant difference in the incidence of pregnancy rate after 5 years of follow-up between women treated with GnRHa at the time of chemotherapy (cyclophosphamide in 67% of women) compared to controls, with age and cumulative dose of cyclophosphamide (> 5 g/m^2^) being better predictors of premature ovarian failure than GnRHa use [95]. Use of surrogate markers of fertility (with pregnancies occurring in patients with protocol-defined premature ovarian failure [95]), and inadequate follow-up of both pregnancy intent and outcome are possible contributors to inconsistency in published data. In the context of conflicting evidence, the American Society of Clinical Oncology recommends that LHRHa/GnRHa may be offered to patients in the hope of reducing the likelihood of chemotherapy-induced ovarian insufficiency when proven fertility preservation methods such as oocyte or embryo cryopreservation are not feasible [96].

Age, anticipated cyclophosphamide dose and patient preference should inform fertility preservation in women with CKD. Whether the assessment of ovarian reserve by serum anti-Mullerian hormone concentrations has clinical utility in predicting benefit from fertility preservation remains unknown.

As cyclophosphamide exposure is a recognized predisposing factor for infertility, a referral for fertility assessment can be made before one year of regular unprotected intercourse, particularly in women with CKD who are aged 36 and over, according to national guidance [90].

##### Guideline 3.2.3

We suggest women with CKD are referred for pre-pregnancy counselling before receiving assisted reproduction (2D).

**Rationale**

Women with CKD considering pregnancy should be offered pre-pregnancy counselling by an expert multidisciplinary team (see Section 3.3). Healthcare providers should recognise that discussions of fertility and referrals for fertility assessment provide an opportunity for expert pre-pregnancy counselling in women with CKD.

##### Guideline 3.2.4

We recommend single-embryo transfer is performed to reduce risk of complications associated with multifetal pregnancies in women with CKD (1C).

**Rationale**

A small case-control study of 15 twin pregnancies in women with CKD shows a higher risk of preterm delivery, growth restriction, neonatal unit admission, weight discordance, perinatal mortality and neonatal mortality compared to both low-risk twin pregnancies and twin pregnancies complicated by either chronic hypertension or collagen disease [97]. This generates a difficult ethical balance between an increased risk of adverse pregnancy outcome due to multifetal pregnancy and the likely success of implantation. There was unanimous consensus amongst the guideline committee that avoidance of iatrogenic twinning with single embryo transfer in CKD patients is safer with regard to maternal-fetal outcomes and should be recommended. It is also worthy of note that in available case series [97], three of the six patients who underwent assisted fertilization were diagnosed with CKD during pregnancy, suggesting that urinalysis and eGFR quantification should be performed as part of the evaluation for assisted fertilization.

#### Pre-pregnancy counseling and optimization for pregnancy

##### Guideline 3.3.1

We suggest women with CKD considering pregnancy are offered pre-pregnancy counselling by a multidisciplinary team including a consultant obstetrician and nephrologist or expert physician (2D).

##### Guideline 3.3.2

We recommend women with CKD are advised there is an increased risk of complications in pregnancy including pre-eclampsia, preterm birth, fetal growth restriction, and neonatal unit (NNU) admission, and that they are more likely to require caesarean delivery (1C).

**Rationale**

Cohort studies [98–100] and meta-analysis [101, 102] show that women with CKD have an increased risk of antenatal complications including pre-eclampsia, preterm delivery, fetal growth restriction compared to women without CKD, although a successful pregnancy is feasible for most women. A meta-analysis that compared 2682 pregnancies in women with CKD with 26,149 pregnancies in healthy controls showed that weighted averages of adverse maternal events in women with CKD and healthy controls were 11.5 and 2% respectively, with a two-fold increase in adverse neonatal outcomes (premature births, fetal growth restriction, small for gestational age, neonatal mortality, stillbirths, and low birth weight) in women with CKD [101]. The likelihood of adverse outcomes are predominantly dependent on baseline excretory renal function, hypertension, proteinuria and, to a lesser extent, aetiology of renal disease [98, 99, 103, 104]. However, as adverse outcomes are more common even in women with preserved excretory renal function (pre-pregnancy CKD stages 1 and 2) than the general obstetric population, counselling should be offered to all women with CKD [98]. A questionnaire study in the UK found that over 90% of women with CKD attending pre-pregnancy counselling found consultations informative and helpful in making a decision on pursuing pregnancy [105].

The provision of pre-pregnancy counselling is likely to depend upon local availability of expertise. However the guideline committee recommend expert, multidisciplinary pre-pregnancy counselling for women with an eGFR < 60 ml/min/1.73m^2^, women with CKD progression, women with uncontrolled hypertension (> 140/90 mmHg), women with nephrotic-range proteinuria, women with active renal disease, women with lupus nephritis, women with renal transplants and all women with previous adverse obstetric outcomes.

##### Guideline 3.3.3

We recommend women with known or suspected inheritable renal diseases are offered genetic counselling including inheritance risk, prognosis, and intervention options including pre-implantation genetic diagnosis (1C).

**Rationale**

Genetic counselling is indicated for families with a history of known or suspected inheritable renal disease to assist in decision-making regarding pursuing a pregnancy. Referral for specialist counselling with clinical genetics teams may be indicated to facilitate genetic diagnosis, the testing of family members or for discuss regarding the possibility of pre-implantation genetic diagnosis (PGD). PGD is approved by the Human Fertilisation and Embryology Authority for autosomal dominant and recessive forms of polycystic kidney disease, Alport syndromes, Fabry disease and Cystinosis [106] and this option might be a consideration for some families.

##### Guideline 3.3.4

We recommend pre-pregnancy counselling for the optimisation of maternal and neonatal outcomes in women with CKD, which may include:

- stabilising disease activity in advance of pregnancy on minimised doses of pregnancy-appropriate medications (1B).
- optimising blood pressure control (< 140/90 mmHg) on pregnancy-appropriate medications (1B).
- optimising glycaemic control in women with diabetes mellitus (1A) (see section 5.4).
- minimising risk of exposure to teratogenic medications (1C) (see section 2).
- making a treatment plan in the event of hyperemesis or disease exacerbation/relapse during pregnancy (1D).

**Rationale**

In addition to specialist renal care, pre-pregnancy advice for women with CKD should follow advice available from the National Institute for Health and Care Excellence to promote optimal long and short-term health outcomes of all women and their children during and after pregnancy [107].

There are observational data that associate active lupus nephritis, nephrotic syndrome and small vessel vasculitis are associated with increased risks of adverse pregnancy outcomes including fetal demise [108–110]. These data and others report more favourable outcomes in women with quiescent disease at the time of conception [111]. Although longitudinal patient data are not available to confirm that disease stabilisation improves pregnancy outcomes, the aim of disease quiescence prior to conception is recommended.

Hypertension is a recognised risk factor for progression of CKD. Non-pregnant women with CKD should therefore be treated according to up to date blood pressure targets. In addition, prospective cohort studies of preconception hypertension show an association with pregnancy loss [112, 113].

##### Guideline 3.3.5

We recommend women with CKD who are taking angiotensin converting enzyme inhibitors have a plan for discontinuation/conversion guided by the strength of indication for renin-angiotensin blockade and the likelihood of pregnancy confirmation in the first trimester (1B).

##### Guideline 3.3.6

We recommend angiotensin receptor antagonists are discontinued in advance of pregnancy (1D).

**Rationale**

Angiotensin converting enzyme inhibitors (ACEi) and angiotensin receptor antagonists are fetotoxic in the second and third trimesters. Exposure to ACEi in the second and third trimester may lead to major birth defects including renal agenesis, and should be avoided. Although retrospective cohort studies show an apparent increased rate of congenital malformation associated with first trimester exposure to ACEi [114], such association is lost after adjustment for confounding factors including hypertension, diabetes, age, obesity and parity [23, 115]. In the largest published cohort, which included 2626 exposed pregnancies, adjusted relative risks associated with first-trimester ACEi exposure compared to unexposed pregnancies were 0.89 (95% CI 0.75–1.06) for overall malformations, 0.95 (95% CI 0.75–1.21) for cardiac malformations, and 0.54 (95% CI 0.26–1.11) for central nervous system malformations [115].

To avoid the risk of inadvertent second trimester exposure to ACEi, these agents can be stopped prior to pregnancy, or as soon as pregnancy is confirmed in women with a strong indication for continued renin-angiotensin blockade during the unknown period of time taken to conceive, such as proteinuric renal disease. Women who continue to take ACEi during attempts to conceive need to be counselled to perform regular pregnancy tests, at least monthly.

There are limited data addressing the risks of exposure to angiotensinogen receptor antagonists in the first trimester. Limited reports of harm [116] and inadequate evidence of safety mean that first trimester exposure to angiotensin receptor antagonists should be avoided. Hence angiotensin receptor blockers should be stopped or substituted before contraception is discontinued.

##### Guideline 3.3.7

We suggest women with CKD stages 4 and 5 contemplating pregnancy are offered pre-dialysis education (2D).

**Rationale**

Observational data from the 1970s identified that women commencing pregnancy with advanced CKD had a 1 in 3 risk of requiring dialysis within a year of the pregnancy. Cohort studies from the 1980s, 1990s and 2000s continue to describe a 1 in 3 risk of dialysis for patients whose serum creatinine approximates to pre-pregnancy CKD stage 4 and 5 [98, 103, 104, 117]. Education about kidney failure and the possibility of antenatal or post-partum dialysis initiation, including treatment options, modality choice (see section 5.2) and access, is therefore recommended prior to conception in line with recommendations for non-pregnant patients approaching dialysis [118].

### Pregnancy Care

#### Assessment of renal function in pregnancy

##### Guideline 4.1.1

We recommend renal function in pregnancy is assessed using serum creatinine concentrations as estimated GFR (eGFR) is not valid for use in pregnancy (1C).

**Rationale**

Due to increased plasma flow and dynamic changes in filtration fraction during pregnancy [119], glomerular filtration increases up to 50% with a consequent fall in serum creatinine concentrations [120]. Analysis of cross sectional serum creatinine concentrations from 243,534 pregnant women in Ontario, Canada defined mean serum creatinine as 60 μmol pre-pregnancy, falling to a nadir of 47 μmol between 16 and 32 weeks’ gestation, peaking at 64 μmol within the first post-partum weeks, before returning to pre-pregnancy concentrations by 18 weeks post-partum. The 95th centile values for serum creatinine were 78 μmol prior to pregnancy, 59 μmol during the second trimester, and 84 μmol in the post-partum period [121]. Meta-analysis of serum creatinine values in pregnancy suggests that the upper reference limits for serum creatinine in pregnancy are 85, 80 and 86% of non-pregnant reference values in the first, second and third trimesters respectively [122].

Estimated glomerular filtration rate (eGFR) derived by both Modified Diet in Renal Disease (MDRD) [123, 124] and Chronic Kidney Disease Epidemiology Collaboration (CKD-EPI) [125] equations have been compared with formal assessment of glomerular filtration rate (GFR) quantified with inulin and found underestimate formal GFR by up to 20% in pregnancy, thus cannot be used. In addition, the dynamic nature of gestational and immediate post-partum change in renal function means that steady state cannot be presumed, prohibiting the use of eGFR. Quantification of GFR by creatinine clearance in pregnancy is unreliable and impractical [124]. Alternative markers of glomerular filtration have not been extensively studied; however cystatin-C has been demonstrated to rise in the second trimester despite a fall in GFR suggesting that additional gestational factors modify renal handling of cystatin-C in pregnancy, preventing utility in the assessment of renal function [126, 127].

##### Guideline 4.1.2

We recommend women with CKD have formal quantification of proteinuria in pregnancy (1D).

**Rationale**

The amount of protein excreted into urine increases in normal pregnancy as a consequence of physiological changes in the kidney with gestation. These changes include an increase in renal blood flow with a corresponding increase in glomerular filtration, a more porous glomerular basement membrane, and altered tubular reabsorption. The amount of protein excreted by the kidney in pregnancy is greater than that in the non-pregnant population. The 95% confidence interval for 24 h urinary protein excretion in 270 healthy pregnant women was found to be 259.4 mg [128], hence abnormal proteinuria is defined as proteinuria levels of > 300 mg/24 h, twice the normal limit in non-pregnant women. For women with CKD, renal adaptation to pregnancy and relative change in proteinuria are not predictable. Formal quantification of proteinuria is therefore required in order to be able to assess relative change in pregnancy, particularly after 20 weeks’ gestation when pre-eclampsia may develop (see sections 4.4.5 and 4.4.6), and in conditions where an increase in proteinuria may represent disease flare or progression.

Proteinuria in early pregnancy also predicts adverse fetal and maternal outcomes in women with CKD. The Torino-Cagliari Observational Study compared obstetric and renal outcomes in 504 women with CKD with 836 women without CKD. Proteinuria (> 1 g/24 h) was an independent risk factor for preterm birth before 37 weeks’ gestation (odds Ratio (OR) 3.65; 95% confidence interval (CI): 1.61–8.24) and 34 weeks’ gestation (OR 4.81; 95% CI 1.48–15.66) [98]. Adverse outcomes associated with proteinuria were corroborated in a systematic review and meta-analysis of 23 studies including 621 pregnancies in women with CKD [102]. This study showed that women with macroproteinuria (albuminuria ≥300 mg/24 h or proteinuria ≥500 mg/24 h) had an increased risk of pre-eclampsia (OR 13.76; 95% CI 8.02–23.63) and preterm birth (OR 5.19; 95% CI 3.21–8.40).

##### Guideline 4.1.3

We recommend quantification of proteinuria is undertaken by protein:creatinine ratio (uPCR) or albumin:creatinine ratio (uACR). Twenty-four hour urine collection for quantification of protein is not required (1B).

**Rationale**

Dipstick testing of urine with reagent strips to detect proteinuria preferentially detects albumin. False positives occur with dehydration, exercise, infection and alkaline urine. False negatives occur with dilute urine, and non-albumin proteinuria. A systematic review of seven prospective studies showed that the sensitivity and specificity of a dipstick result of ≥1+ protein for predicting abnormal proteinuria in pregnancy (> 300 mg/24 h) ranges from 47 to 86% and 39–95% respectively, leading to the conclusion that the accuracy of dipstick urinalysis with a 1+ threshold in the prediction of significant proteinuria is poor [129]. However, automated dipstick urinalysis provides a more accurate screening test for the detection of proteinuria than visual testing in hypertensive pregnancies [130].

24-h urine collection is time-consuming and subject to inadequacies in collection [131]. Outside of pregnancy, uPCR and uACR are highly correlated with 24-h urine collection and are more convenient in clinical practice [132]. Pregnant cohorts show a similar correlation between 24-h urine protein excretion and both uPCR [133] and uACR [134]. A prospective multi-centre cohort study of 959 pregnant women after 20 weeks’ gestation with hypertension and trace protein or more on urine dipstick found that both uPCR and uACR could be both used as rule out tests for preeclampsia with no additional benefit from 24-h urine collection [135].

There is on-going debate as to whether uPCR or uACR should be preferentially used for the quantification of proteinuria in pregnancy. In non-pregnant patients with CKD, uACR is the investigation of choice as it provides greater sensitivity at lower levels of proteinuria, although uPCR can be used as an alternative, particularly where uACR is 70 mg/mmol or greater [136]. In contrast, uPCR is currently the most common test used to quantify proteinuria in pregnancy [137]. A single centre experience of 181 pregnant women without CKD showed that uACR and uPCR were highly correlated to each other, with equivalent performance in the prediction of adverse pregnancy outcomes [138]. More recent, larger, prospective cohort data from normal pregnancies show that although uACR and uPCR are comparable in performance, uACR had a significantly higher area under the receiver-operating curve (ROC) for the diagnosis of severe pre-eclampsia compared to local laboratory uPCR (ROC 0.89 versus 0.87, *p* = 0.004). However it remains unclear whether this small absolute difference translates into significant clinical benefit. Cost effectiveness for uACR over uPCR was also suggested, although 95% confidence intervals for the incremental cost-effectiveness ratio crossed zero due to significant uncertainty and the small difference in incremental cost and quality added life years [135]. There are no published data on the predictive and/or diagnostic benefits of uACR compered to uPCR in pregnant women with CKD. It is therefore the consensus of the guideline group that the decision to use uACR or uPCR should be based on local obstetric experience ensuring a baseline measurement in early pregnancy in order to be able to recognise relative change in proteinuria in pregnancy. In women without pre-existing proteinuria, diagnostic performance equivalent to 30 mg/mmol of uPCR is achieved with a uACR cut-off of 8 mg/mmol [135].

#### Antenatal care

##### Guideline 4.2.1

We suggest pregnant women with CKD who have not had pre-pregnancy counselling by the MDT are referred to the MDT and receive the same counselling and optimisation as for women attending pre-pregnancy (2D).

##### Guideline 4.2.2

We recommend pregnant women with CKD receive routine antenatal care, in addition to specialist input (1D).

##### Guideline 4.2.3

We recommend pregnant women with CKD be referred for assessment by a consultant obstetrician (1D).

##### Guideline 4.2.4

We recommend pregnant women with CKD have access to usual trisomy screening with specialist interpretation of high-risk results (1C).

##### Guideline 4.2.5

We recommend women with CKD exposed to teratogenic drugs in the first trimester are referred to a specialist fetal medicine unit (1D).

##### Guideline 4.1.6

We recommend pregnant women with CKD have scans to assess fetal growth and wellbeing in the third trimester (1C).

##### Guideline 4.2.7

We recommend pregnant women taking prednisolone and/or calcineurin inhibitors are screened for gestational diabetes (1C).

**Rationale**

Women with CKD who present for the first time in pregnancy should have an opportunity for individualised risk counselling and optimisation of health for pregnancy. These women should therefore be referred to the MDT as early as possible in pregnancy to ensure that the same topics are covered as for women receiving pre-pregnancy counselling (see section 3.3). This reflects lessons learned from MBRRACE-UK (Mothers and Babies: Reducing Risk through Audit and Confidential Enquiries across the UK), which has identified maternal medical comorbidities to be significantly associated with maternal morbidity and mortality [139, 140].

There is no specific literature on the schedule of care for women with CKD. Women with CKD should be supported to access routine antenatal care, with increased surveillance in line with national guidance for antenatal care [141], and guidance on the management of hypertension in pregnancy [14]. A personalised care plan including a named midwife should be made [142], ensuring access to the specialist MDT. It is good practice that a consultant obstetrician reviews all women with CKD to help ensure that the most appropriate care pathway is identified.

Pathways for care for women with CKD in pregnancy should map to those agreed for the regional Maternal Medicine Network and Maternal Medicine Centre. If the Maternal Medicine Centre and the regional renal unit are not co-located (which was the case for 31% of responders to the consensus survey undertaken for this guideline) then the consultant obstetrician and consultant nephrologist should communicate regularly during the pregnancies of women with CKD, ensuring access to all notes and results.

Women with CKD should be offered usual trisomy screening. If they have abnormal renal function, the pre-test counselling for combined screening using blood markers should include discussion of a potentially increased false positive rate as the multiple of the median may be increased for beta human chorionic gonadotrophin, though not for pregnancy associated plasma protein-A [143]. Other screening options such as the non-invasive prenatal testing of cell-free fetal DNA, either as a first line or subsequent to combined screening, currently depend upon local availability. Women with CKD and abnormal renal function should be referred to a specialist Fetal Medicine Unit for interpretation of positive results.

The care pathway should include determination of frequency of third trimester ultrasound for evaluation of fetal growth based on a woman’s individualised risk assessment for fetal growth problems. In light of the known association between steroids and calcineurin inhibitors with impaired glucose metabolism [144], screening for gestational diabetes should be arranged for women taking these medications.

Options for maternity care, determined by the MDT, include:

- Advice regarding pregnancy care and delivery, with referral back to the local maternity unit
- Shared maternity care between the Maternal Medicine Centre and the local unit
- Maternal Medicine Centre to lead and deliver maternity care

#### Pre-eclampsia prophylaxis

##### Guideline 4.3.1

We recommend women with CKD are offered low-dose aspirin (75-150 mg) in pregnancy to reduce the risk of pre-eclampsia (1B).

##### Guideline 4.3.2

We suggest kidney donors are offered low dose aspirin (75 mg–150 mg) to reduce the risk of pre-eclampsia (2D).

**Rationale**

Women with CKD have an increased risk of pre-eclampsia compared to women without CKD [99] and should be offered pre-eclampsia prophylaxis with aspirin. This recommendation is generalised from high-quality evidence that low dose aspirin is associated with a reduced incidence of pre-eclampsia in other high-risk cohorts [8, 9], although there is limited definitive evidence as to the optimal gestation and dose for women with CKD. A recent subgroup analysis of women with chronic hypertension from a randomised-controlled trial of 150 mg aspirin (150 mg) failed to show a reduction in the risk of subsequent pre-eclampsia, although these data are difficult to interpret in the absence of standardised diagnostic criteria for superimposed pre-eclampsia. Current national guidance recommends prescribing 75-150 mg of aspirin from 12 weeks’ gestation onwards [9, 14], but future research may elucidate optimisation of prophylaxis in women with CKD.

Women who have donated a kidney are at increased risk of pre-eclampsia (odds ratio 2.4; 95% confidence intervals 1.0–5.6) [145]. Pre-eclampsia prophylaxis with aspirin should be discussed with these women, particularly in the presence of other known risk factors as outlined in national guidelines [14].

The benefit of calcium supplementation in reducing the prevalence of pre-eclampsia remains unclear. A Cochrane systematic review of randomised controlled trials showed that supplementation of a least 1 g calcium per day was associated with a 55% reduction in pre-eclampsia, although the effect was mostly shown in smaller trials, with possible confounding by low dietary calcium intake [146]. In contrast, large randomised controlled trials of calcium supplementation starting both before [147] and after 20 weeks’ gestation [148] have failed to show a benefit in reducing the incidence of pre-eclampsia. In the absence of evidence specific to women with CKD, and given the potential cardiovascular sequelae of a positive calcium balance in women with CKD [149, 150], it was the consensus opinion of the guideline committee that calcium supplementation to reduce the risk of pre-eclampsia cannot be recommended for women with CKD, based on current evidence.

#### Blood pressure management

##### Guideline 4.4.1

We recommend that the target blood pressure during pregnancy for women with CKD is 135/85 mmHg or less, which should be documented in the woman’s healthcare record (1D).

##### Guideline 4.4.2

We suggest antihypertensive treatment in women with CKD is continued in pregnancy unless systolic blood pressure is consistently < 110 mmHg systolic, or diastolic blood is pressure consistently < 70 mmHg diastolic BP, or there is symptomatic hypotension (2D).

##### Guideline 4.4.3

We recommend labetalol, nifedipine and methyldopa can be used to treat hypertension in pregnancy (1B).

##### Guideline 4.4.4

We recommend angiotensin converting enzyme inhibitors, angiotensin receptor anatgonists and diuretics are not used to treat hypertension in pregnancy (1B).

##### Guideline 4.4.5

We recommend a diagnosis of superimposed pre-eclampsia is considered:

- in a woman with non-proteinuric CKD, if she develops new hypertension (systolic BP > 140 mmHg and/or diastolic BP > 90 mmHg) and proteinuria (uPCR > 30 mg/mmol or uACR > 8 mg/mmol) or maternal organ dysfunction after 20 weeks’ gestation (1B).
- in a women with proteinuric CKD if she develops new hypertension (systolic BP > 140 mmHg and/or diastolic BP > 90 mmHg) or maternal organ dysfunction after 20 weeks’ gestation (1B)
- in a women with chronic hypertension and proteinuria, if she develops maternal organ dysfunction after 20 weeks’ gestation (1B).

##### Guideline 4.4.6

We suggest in women with chronic hypertension and proteinuria that the development of sustained severe hypertension (systolic BP > 160 mmHg and/or diastolic BP > 110 mmHg or doubling of antihypertensive agents) and/or a substantial rise in proteinuria (doubling of uPCR or uACR compared to early pregnancy) should prompt clinical assessment for superimposed pre-eclampsia (2D).

##### Guideline 4.4.7

We suggest a role for angiogenic markers (PlGF±sFlt-1) is considered as an adjunct to diagnose superimposed pre-eclampsia, dependent upon on-going research in women with CKD (2C).

**Rationale**

There is no evidence on treatment initiation thresholds or blood pressure targets in pregnancy for women with CKD. Randomised controlled trial data from women with non-proteinuric hypertension demonstrate that tight blood pressure control (aiming for a diastolic BP of 85 mmHg) reduces severe maternal complications, with no evidence of perinatal harm [151]. Evidence from systematic reviews has shown that beta blockers (such as labetalol) and calcium channel blockers (such as nifedipine) appear to be more effective than methyldopa in avoiding an episode of severe hypertension (RR 0.70; 95% CI 0.56 to 0.88; 11 trials, *n* = 638) [152]. Antihypertensive agents such as ACE inhibitors, angiotensin receptor blockers and diuretics should be avoided in pregnancy due to potential for fetal harm (see section 2). In women who have been maintained on these antihypertensive agents whilst waiting to conceive, they should be switched to labetalol or nifedipine (or a suitable alternative) within two days of notification of pregnancy [14].

Making the diagnosis of superimposed pre-eclampsia is complex in women with chronic kidney disease, particularly in the presence of pre-existing proteinuria and/or hypertension as these two signs are part of the diagnostic criteria for pre-eclampsia. Appearance of a new feature and/or the development of maternal organ dysfunction (Table 2) should lead to a diagnosis of preeclampsia being considered. Proteinuria is usually a feature of pre-eclampsia; however it is not required for diagnosis where there is other maternal organ dysfunction [137]. Although it is recognized that gestational rises in blood pressure or protein excretion may occur, sudden and substantial change in these parameters in a woman with CKD should prompt her clinicians to evaluate her for a diagnosis of superimposed pre-eclampsia.

**Table 2:** Maternal organ dysfunction in pre-eclampsia. Adapted from [137]

• New proteinuria (uPCR> 30 mg/mmol or ACR > 8 mg/mmol)	
• AKI (serum creatinine ≥90 μmol/L in a woman with previously normal creatinine concentrations)	
• Liver involvement (alanine aminotransferase or aspartate aminotransferase > 40 IU/L) with or without right upper quadrant or epigastric pain	
• Neurological complications (eclampsia, altered mental status, blindness, stroke, clonus, severe headache, persistent visual scotomata)	
• Hematological complications (platelet count < 150,000/μL, disseminated intravascular coagulation, hemolysis)	
• Uteroplacental dysfunction (fetal growth restriction, abnormal umbilical artery Doppler wave form analysis, stillbirth	

National guidelines recommend the use of placental growth factor-based testing (e.g. Triage placental growth factor (PlGF) test or the Elecsys immunoassay soluble fms-like tyrosine kinase 1 (sFlt-1)/PlGF ratio), alongside standard clinical assessment and subsequent clinical follow-up, to help rule-out pre-eclampsia in women presenting with suspected pre-eclampsia between 20 weeks and 34 weeks plus 6 days of gestation [153]. These tests have high sensitivity and negative predictive value for the diagnosis of pre-eclampsia and the need for delivery within 14 days in general obstetric cohorts [154, 155]; use of revealed PlGF testing halves the time to diagnosis of pre-eclampsia and reduces severe maternal adverse outcomes [156]. PlGF-based testing has been reported to have similar diagnostic utility in small cohorts of women with CKD [100, 157].

#### Venous thromboembolism

##### Guideline 4.5.1

We recommend that women with nephrotic-range proteinuria (uPCR> 300 mg/mmol or ACR > 250 mg/mmol) be offered thromboboprophylaxis with low molecular weight heparin in pregnancy and the post-partum period unless there is a specific contraindication including risk of labour or active bleeding (1D).

##### Guideline 4.5.2

We suggest that non-nephrotic range proteinuria in pregnancy is a risk factor for thrombosis and thromboprophylaxis with low molecular weight heparin should be considered in the presence of additional risk factors (2D).

**Rationale**

The guideline committee endorses the assessment and management of VTE risk in women with CKD according to guidance from the Royal College of Obstetricians and Gynaecologists (RCOG) which states that all pregnant women undergo a documented assessment of risk factors for venous thromboembolism [10]. Nephrotic syndrome is included as a significant risk factor in RCOG guidance with thromboprophylaxis offered to women with nephrotic syndrome in the third trimester and post-partum in the absence of other risk factors. However, there is inherent difficulty in making the diagnosis of nephrotic syndrome in pregnancy as physiological adaptation to pregnancy includes increased proteinuria, a fall in serum albumin concentrations, and peripheral oedema. Gestation specific thresholds for proteinuria and serum albumin for the diagnosis of nephrotic syndrome in pregnancy have not been established. This guideline therefore opts to use the Renal Association threshold for nephrotic range proteinuria (uPCR> 300 mg/mmol or ACR > 250 mg/mmol) to define high-risk proteinuria in pregnancy for which there is expert consensus that thromboprophylaxis is warranted in pregnancy and the post-partum period in the absence of other risk factors (with risk reassessed at 6 weeks post-partum).

There is also consensus, but insufficient evidence, that sub-nephrotic levels of proteinuria confer a risk of thrombosis although the threshold level of proteinuria at which the risk of VTE is clinically significant remains unknown. Consequently, thromboprophylaxis in women with CKD varies across the UK and internationally. There are anecdotal data that regions with a higher threshold for thromboprophylaxis do not report many/any thrombotic events attributable to proteinuria alone. In contrast, clinicians with a lower threshold for recommending thromboprophylaxis accept the compromise that maternal morbidity and mortality from thrombosis justifies the number needed to treat. In the light of this uncertainty, the guideline committee suggest that non-nephrotic range proteinuria (uPCR> 100 mg/mmol or uACR> 30 mg/mmol) be considered a risk factor for thrombosis and thromboprophylaxis with low molecular weight heparin be offered in the presence of additional risk factors. Recognised risk factors include gestation, other medical comorbidity (including active lupus), age, BMI, parity, smoking, gross varicose veins, pre-eclampsia, assisted reproduction, operative delivery, post-partum haemorrhage, still-birth, hyperemesis, infection, and immobility [10]. Renal disease aetiology, the trajectory of proteinuria and serum albumin concentrations may also inform the decision to offer thromboprophylaxis, although specific guidance on these factors is not possible due to insufficient evidence.

#### Anaemia

##### Guideline 4.6.1

We recommend pregnant women with CKD are given parenteral iron if indicated (1C).

##### Guideline 4.6.2

We recommend erythropoietin stimulating agents are given if indicated in pregnancy (1C).

**Rationale**

Gestational increases in plasma volume are higher than the corresponding increase in red blood cell mass, leading to haemodilution and lower haemoglobin levels in pregnancy. The lower reference limit for haemoglobin concentrations in pregnancy is 105 g/L-110 g/L depending on gestation [158, 159], with values < 85 g/L associated with an estimated 62% increase in the risk of low birth weight (< 2500 g) and a 72% increase in the risk of preterm delivery before 37 weeks, across ethnic groups [160]. There are no data to guide the optimum target haemoglobin for women with CKD in pregnancy.

The most common cause of anaemia in pregnancy is iron deficiency, which is estimated to affect greater than 40% of pregnancies [161]. Markers of iron deficiency include ferritin (< 100 μg/L), transferrin saturation (< 20%), hypochromic red cells (> 6%) and reticulocyte haemoglobin content (< 25 pg), although the specificity and sensitivity of these markers in pregnancy are unknown [162]. Oral iron is cheap and accessible, although the intravenous route may offer better bioavailability and tolerability in pregnancy, in women with CKD [55]. Parenteral iron is considered safe in pregnancy and breastfeeding [56–59], although there is a paucity of safety data on exposure in the first trimester.

Erythropoietin concentrations increase approximately two-fold during pregnancy [60]. As women with CKD may have insufficient capacity for a gestational increase in erythropoietin, supplementation with synthetic erythropoietin may be required, even in the context of mild or moderate renal impairment. For women who required erythropoietin prior to pregnancy, an increased dose requirement should be anticipated during pregnancy. As erythropoietin is a large molecule that does not cross the placental barrier, its use is considered safe in pregnancy and breastfeeding [61, 62]; however, a theoretical risk of exacerbating pre-existing or new-onset hypertension exists.

Hypoxia-inducible factor (HIF) activators are an emerging class of drugs with a therapeutic role in the management of renal anaemia. However, the small size of these molecules potentially enables placental transfer, and HIF has multiple direct and indirect effects on developmental and physiological processes [163]. No formal recommendation has been made for the use of this class of drug in pregnancy, but on the basis of their molecular characteristics, the guideline committee would not recommend their use at conception or in pregnancy and lactation.

#### Bone health

##### Guideline 4.7.1

We recommend women with CKD who are vitamin D deficient be given vitamin D supplementation in pregnancy (1B).

##### Guideline 4.7.2

We recommend calcimimetics are discontinued in pregnancy (1D).

##### Guideline 4.7.3

We recommend non-calcium based phosphate binders are discontinued in pregnancy (1D).

**Rationale**

Vitamin D deficiency is estimated to affect 13–64% of pregnant women [164] and is associated with increased incidences of pre-eclampsia and gestational diabetes. Although studies of the benefit of vitamin D on pregnancy outcome are inconsistent, meta-analysis demonstrates that oral vitamin D supplementation is associated with reduced risks of pre-eclampsia, low birth weight and preterm birth [165, 166]. Optimal serum calcifediol (25(OH)-vitamin D) levels and optimal doses of colecalciferol and ergocalciferol are unknown. It is the clinical practice of the guideline committee to check serum calcifediol levels in pregnancy, and offer replacement (colecalciferol 20,000iu per week) until serum calcifediol is > 20 ng/ml (> 50 nmol/L). Although calcifediol is the major circulating form of vitamin D, it has low biological activity until converted to calcitriol (1,25(OH)_2_-vitamin D). Serum calcitriol levels are approximately threefold higher in the first trimester and 5–6 times higher in the third trimester compared with those in non-pregnant women [167]. To what extent this increase is dependent on the 1α-hydroxylase enzyme activity in the kidney is unknown^,^ as the enzyme is also found in colon, skin, macrophages and the placenta. In the absence of better evidence, the guideline committee suggests that, once serum calcifediol levels are replete, activated vitamin D analogues (alfacalcidol, calcitriol) can be continued in pregnancy at a dose that would be considered appropriate for maintenance treatment outside of pregnancy. For women with CKD who do not require activated analogues, a maintenance daily dose of vitamin D 400-1000iu can be given in pregnancy, depending on ethnicity and body mass index.

Calcimemtics (cinecalcet, etelcalcitide) and non-calcium phosphate binders (sevelamer hydrochloride, lanthanum carbonate) have insufficient safety data in pregnancy and should therefore be discontinued in advance of pregnancy and during lactation.

#### Renal biopsy

##### Guideline 4.8.1

We recommend if a histological diagnosis will change management in pregnancy then renal biopsy can be performed in the first and early second trimester of pregnancy (1C).

**Rationale**

Published data on antenatal renal biopsy are limited by heterogeneity and cohort size. The most commonly described risk is bleeding. Contributory factors are thought to include the increased renal blood flow in pregnancy and the technical difficulty in performing a renal biopsy in the standard prone position at later gestations. A systematic review on renal biopsy in pregnancy including 39 studies published between 1980 and 2012, examined 243 antenatal biopsies compared with 1236 postpartum biopsies [168]. This showed that the risk of renal biopsy complications was significantly higher in antenatal biopsies compared with those postpartum (7% vs 1%, *p* = 0.001). Complications, including macroscopic haematuria, perirenal haematomata, and the need for blood transfusion were described between 23 and 28 weeks’ gestation. No serious complications occurred prior to 22 weeks’ gestation.

Women who undergo renal biopsy in pregnancy have histological diagnoses spanning the spectrum of glomerular disease [169], although treatment options may be limited in pregnancy due to the teratogenicity and/or fetal toxicity of available treatments [170]. A change in management based on the results of a renal biopsy in pregnancy was reported in 39/59 (66%) of women [168].

The decision to undertake renal biopsy in pregnancy must balance the increased risk of bleeding, the likelihood of a change in management based on the biopsy result, and the risks of either iatrogenic preterm delivery or a delay in management until a biopsy can be performed post-partum. It is the consensus of the guideline group that renal biopsy in pregnancy should be performed by the most experienced clinician available, under ultrasound guidance.

#### Peripartum care

##### Guideline 4.9.1

We recommend women with CKD receive routine peripartum care, with additional specialist input (1D).

##### Guideline 4.9.2

We recommend women with CKD have observations taken and documented during any hospital admission. This includes temperature, heart rate, blood pressure, respiratory rate, and oxygen saturation. An early warning score should be calculated and actioned appropriately (1D).

##### Guideline 4.9.3

We recommend additional assessment for women with an elevated early warning score, for women considered to be high-risk, and for any women in whom there is any clinical concern. This includes examination of jugular venous pressure, lung auscultation and urine output monitoring (in-dwelling catheter not usually required) in addition to routine parameters (1D).

**Rationale**

The guideline committee endorses existing guidelines on Intrapartum Care for Healthy Women and Babies [171], Intrapartum Care for Women with Existing Medical Conditions or Obstetric Complications and their Babies [172], and recommendations from the Royal College of Anaesthetists on the care of the critically ill pregnant woman [173].

Failure to recognise the signs of illness in obstetric patients is a recurrent feature of cases of maternal morbidity and mortality [5, 174, 175]. The use of early warning systems is established in non-obstetric acute care settings. Although evidence linking implementation of obstetric early warning scores to improved pregnancy outcomes is limited, recent ethnographic research shows that a modified obstetric early warning score is valuable in structuring the surveillance of hospitalised women with an established risk of morbidity [176]. At present, there are variation in obstetric early warning score thresholds used in the UK and the guideline committee endorses the view of the Royal College of Anaesthetists that there should be a move towards a national early warning system, modified for obstetrics [173]. It is hoped that the on-going Pregnancy Physiology Prediction Pattern study [177] will add valuable, gestation-specific, normal distribution data for physiological parameters in pregnancy, and inform trigger thresholds for future validation. Pregnant women demonstrate a capacity for physiological compensation before a potentially rapid deterioration. It was the consensus opinion of the guideline committee that an elevated early warning score in obstetrics should therefore trigger early senior review. The principle of a maternity warning score is intuitively sound, but should not overrule clinical judgement, hence the recommendation for detailed assessment of any woman in whom there is any clinical concern.

##### Guideline 4.9.4

We recommend women with CKD at risk of volume depletion or volume overload are highlighted by the MDT in advance of delivery (1D).

##### Guideline 4.9.5

We recommend that fluid balance is managed with the aim of maintaining normal fluid volume, avoiding dehydration and pulmonary oedema, with input from clinicians with expertise in fluid balance and renal disease (1D).

##### Guideline 4.9.6

We recommend all clinicians are aware of the increased risk of pulmonary oedema in women with CKD and pre-eclampsia (1D).

**Rationale**

It is the experience of the guideline committee that fluid management for women with CKD is often complex and needs to be tailored to the individual. Women at risk of either volume depletion or fluid overload should be highlighted in advance of delivery to ensure that clinical review of fluid state is undertaken prior to being prescribed intravenous fluids, or the institution of a fluid restriction. On-going fluid balance review should then be performed during labour and in the immediate post-partum period with the aim of euvolaemia and the avoidance of both pulmonary oedema and superimposed kidney injury. Fluid balance assessment should be undertaken by a competent clinician with an understanding of the haemodynamic changes in pregnancy and the puerperium. This may include nephrologists, anaesthetists, obstetric physicians, and maternal medicine specialists.

The guideline committee endorses the NICE guideline on hypertension in pregnancy for the management of pre-eclampsia in pregnancy [14]. The risk of pre-eclampsia is higher in all stages of CKD compared to women without CKD [98]. Pre-eclampsia is complicated by capillary leak, reduced plasma oncotic pressure, and either reduced or increased cardiac output [178–180]. The complexity and dynamic nature of pre-eclampsia mean that there should be a plan in place for regular fluid balance review by a competent clinician with expertise in CKD and pre-eclampsia. The aim of fluid balance in pre-eclamspia is euvolaemia. Insensible losses should then be replaced (30 ml/hr) along with anticipated urinary losses (0.5–1 ml/kg/hr), whilst limiting overall fluid intake to 80–100 ml/hr. to avoid the risk of pulmonary oedema [137].

##### Guideline 4.9.7

We recommend the timing of birth for women with CKD is determined by obstetric indications, with consideration of renal factors including deteriorating renal function, symptomatic hypoalbuminaemia, pulmonary oedema, and refractory hypertension (1D).

**Rationale**

In women with CKD mode and timing of birth is usually determined by obstetric factors. Where maternal complications arise, clinicians must balance the competing risks of preterm delivery and maternal wellbeing. Potential maternal complications, which may inform the decision for iatrogenic preterm delivery in women with CKD, include loss of maternal renal function, symptomatic nephrotic syndrome including pulmonary oedema, and refractory hypertension. If maternal complications develop before 34 weeks’ gestation, attempts should be made to continue the pregnancy if possible, due to the reduction in adverse neonatal and developmental outcomes at gestations of 34 weeks’ gestation and more [181], although this decision will depend upon maternal wellbeing and the availability and likely success of medical management options. The guideline committee acknowledges that the diagnosis of superimposed kidney injury is difficult in pregnancy due to gestational variation in serum creatinine concentrations, possible underlying disease progression, and an unpredictable physiological response to pregnancy in CKD. In women without CKD, serum creatinine concentrations fall to a nadir in the second trimester before rising back towards pre-pregnancy levels at term [121]. The guideline committee therefore suggest that women with serum creatinine concentrations in pregnancy that are greater than pre-pregnancy concentrations warrant discussion with and/or assessment by the MDT.

There is no evidence that mode of delivery affects maternal renal function. Mode of delivery should therefore be based on obstetric indications and maternal preference according to guidance in women without CKD [141].

#### Postnatal care

##### Guideline 4.10.1

We recommend that non-steroidal anti-inflammatories should not be given (1C).

**Rationale**

Given that the risk of renal side effects from short-term use of non-steroidal anti-inflammatory drugs in patients without pre-existing risk factors is considered to be rare, NSAIDs are currently recommended in the postpartum period for perineal pain when paracetamol provides insufficient relief of symptoms [182]. Although the risk profile of non-steroidal anti-inflammatory drugs is considered to be different in CKD, evidence supporting this is mixed. Historical case-control studies [183, 184] show an increased rate of kidney injury and progression to end stage renal disease in patients taking non-steroidal anti-inflammatory drugs. In contrast, data from older-age cohorts taking high-dose NSAIDS are conflicting [185–187], Questionnaire data from a female cohort showed no measurable association between NSAID use and renal function decline over 11 years, although the mean eGFR at study commencement was 88 ml/min/1.73m^2^ [188]. There are no data examining non-steroidal anti-inflammatory drug use in women of reproductive age with risk factors for renal disease progression, in the context of peripartum haemodynamic change. The guideline committee therefore endorses existing recommendations that NSAIDs should be contraindicated in women with a (pre-pregnancy) eGFR < 30mls/min/1.73m^2^ (estimated to be equivalent to serum creatinine > 150 μmol/L in pregnancy) [189, 190], and should be avoided where possible in all those with renal impairment due to the possibility of sodium and water retention and a deterioration in renal function [189].

##### Guideline 4.10.2

We recommend women with CKD have a planned early postpartum renal review (1D).

**Rationale**

There are no published data to guide post-partum surveillance on women with CKD. Whilst guidelines for the management of CKD do not require follow up secondary care for all patients, every woman with suspected kidney disease (acute or chronic) newly identified in pregnancy and all those with known CKD should have a clear plan in place for appropriate postpartum follow up [136, 172, 191]. Timing of postpartum follow-up should be determined by the MDT, guided by the level of and change in renal function, the aetiology of CKD, blood pressure, and the need for post-partum therapeutic drug monitoring. For women who are thought to have previously undiagnosed CKD in pregnancy, post-partum renal review should be arranged in order to facilitate diagnosis. This may not have been possible in pregnancy if a biopsy was not done, or due to difficulties interpreting renal function in the context of gestational change or in the face of superimposed preeclampsia. Appropriate treatment should be advised and a pathway for long-term care made clear both to the patient and her primary care physician. The key is to avoid women being lost to follow-up and presenting years later with what might have been avoidable, progressive kidney disease.

##### Guideline 4.10.3

We recommend that women with CKD are prescribed medications that are compatible with breastfeeding whenever possible (1D).

**Rationale**

Women with CKD should be supported in their wish to breastfeed and be prescribed medications which are considered safe in lactation (see section 2). Currently, breastfeeding is not advised for infants of mothers taking mycophenolate mofetil as there are no data to confirm safety. If mycophenolate mofetil is deemed to be the only therapeutic option, then breastfeeding should be avoided.

##### Guideline 4.10.4

We recommend that women with CKD are offered safe and effective contraception post-partum and receive updated pre-pregnancy counselling before future pregnancies (1D).

**Rationale**

The provision of information and a choice regarding contraceptive method within seven days of delivery has been set as a quality standard in the UK, addressing a priority area for quality improvement in health and social care [192]. Safe and effective methods of contraception in women with CKD are detailed in section 3.1.3.

Events during pregnancy including obstetric complications, the development of superimposed pre-eclampsia, and a decline in maternal renal function will inform future obstetric risk, necessitating the need for new pre-pregnancy counselling to ensure informed decision-making regarding future pregnancy.

### Specific conditions

#### Renal transplantation

##### Guideline 5.1.1

We recommend women with renal transplants wait until their kidney function is stable on medications that are safe in pregnancy before conceiving, which is usually more than one year after transplantation (1D).

**Rationale**

Pregnancy rates are lower in women of reproductive age with renal transplants compared to the general population [193, 194]. It is unclear whether this is due to reduced fertility or patient choice. Women with renal transplants usually have successful pregnancy outcomes, but maternal and neonatal complications remain higher compared to the general population [72]. A prospective UK cohort study of 105 pregnancies in 95 women with renal transplants compared with 1360 healthy controls, showed an increased risk of pre-eclampsia (adjusted odds ratio (aOR) = 6.31), induction of labour (aOR = 2.67) caesarean delivery (aOR = 4.57), preterm delivery < 37 weeks (aOR = 12.57) and < 32 weeks (aOR = 4.15), and small for gestational age babies (aOR = 2.92) [65].

There are few data supporting timing of pregnancy in women with renal transplants. Older reports suggested that a shorter interval from transplant to pregnancy was associated with worse pregnancy outcomes [71, 195]. However, a meta-analysis of studies with mean transplant-to-pregnancy intervals of < 2 years (3 studies), 2–3 years (10 studies), 3–4 years (14 studies) and > 4 years (14 studies) concluded that a shorter time from transplant to conception was associated with a higher live birth rate and lower miscarriage rate, although rates of pre-eclampsia, gestational diabetes, caesarean section, and preterm birth were higher [72]. Impact of pregnancy on graft function related to the interval between transplantation and conception is variably reported. A recent study of Medicare data demonstrated that graft loss was significantly higher in women who conceived within two years of transplantation, but those who waited three years or more were not at greater risk of graft lost than women who did not have pregnancies [196].

European Best Practice Guidelines (2001) recommended a delay of 24 months between transplantation and conception [197], but American guidelines (2005) subsequently advised 12 months if stable graft function [198]. Standard immunosuppression regimens in the UK routinely include mycophenolate mofetil in the first year after transplantation [199]. Due to the teratogenicity of mycophenolate mofetil, switching to alternative agents is recommended before pregnancy (see section 2), thus at least a year after transplantation is advised before attempting to conceive.

Other factors to consider regarding timing of pregnancy include recent episodes of rejection, stability and level of graft function, presence of cytomegalovirus, maternal age, diabetes and blood pressure control, but direct evidence regarding impact of these factors on pregnancy and graft outcomes is limited.

##### Guideline 5.1.2

We recommend that plans for delivery in a woman with a renal transplant are discussed with the local surgical transplant team (1D).

##### Guideline 5.1.3

We recommend that mode of delivery in women with renal transplants is based on obstetric indications and maternal preference (1D).

##### Guideline 5.1.4

We recommend that caesarean delivery in a woman with a renal transplant patient is performed by the most senior obstetrician available, ideally a consultant (1D).

##### Guideline 5.1.5

We recommend that women with kidney-pancreas transplants, kidney-liver transplants, and dual kidney transplants are managed during pregnancy and delivery by a multidisciplinary team including transplant physicians and surgeons, at a transplant centre (1D).

**Rationale**

The majority of women with renal transplants have caesarean deliveries [72]. However, renal transplantation is not a contraindication for vaginal delivery. Caesarean section is associated with increased bleeding risk, thromboembolism, infection, surgical complications (for example, ureteric injury) and renal transplant injury has been reported [200]. Vertical skin incision prior to horizontal uterine incision can theoretically be used to reduce the risk of allograft injury, although there are no data on the relative benefits and long-term outcomes of this technique.

Dual organ transplants are associated with higher rates of adverse pregnancy outcomes [201]. A small cohort study demonstrated increased rates of urinary tract obstruction in women with intraperitoneal grafts [202]. The complex anatomy of dual transplants is such that the guideline committee recommends management and delivery at a transplant centre whenever possible.

#### Dialysis

##### Women receiving maintenance dialysis before pregnancy

**Guideline 5.2.1**

We recommend women established on dialysis prior to pregnancy receive pre-pregnancy counselling including the options of postponing pregnancy until transplantation (when feasible) and the need for long frequent dialysis prior to and during pregnancy (1C).

**Guideline 5.2.2**

We recommend women established on haemodialysis prior to pregnancy receive long, frequent haemodialysis either in-centre or at home to improve pregnancy outcomes (1C).

**Guideline 5.2.3**

We suggest women receiving haemodialysis during pregnancy have dialysis dose prescribed accounting for residual renal function, aiming for a pre-dialysis urea < 12.5 mmol/l (2C).

**Guideline 5.2.4**

We recommend women established on peritoneal dialysis prior to pregnancy should convert to haemodialysis during pregnancy (1D).

**Rationale**

Reported pregnancy outcomes for renal transplant recipients remain better than for dialysis recipients, and advice to wait for a renal transplant prior to pregnancy is appropriate for most women with established end stage renal disease [203].

Evidence for the management of pregnancy in women receiving dialysis is limited to observational cohort studies, and prone to publication bias. Nevertheless, cohort studies and meta-analysis show an association between increased dialysis provision and improved fertility and pregnancy outcomes [66, 204–207]. A cohort of women who received 48 ± 5 h of dialysis per week had a conception rate of 32 pregnancies per 1000 women/year compared with 5 per 1000 women/year in a separate cohort receiving fewer than 20 h of dialysis per week [204, 208]. Dialysis in pregnancy for 37–56 h/week compared to fewer than 20 h/week also resulted in a higher live birth rate (85% versus 48%), higher median gestational age at delivery (38 weeks versus 28 weeks) and a greater median birth weight (2600 g versus 1800 g) [66].

It is acknowledged that achieving 48 ± 5 h/week dialysis is not feasible for many women or dialysis centres in the UK. An alternative approach is an increase in haemodialysis provision guided by biochemical parameters. A retrospective observational study of 28 pregnancies in women on haemodialysis compared successful pregnancies in which the baby survived to one year with unsuccessful pregnancies. Despite no overall difference in weekly dialysis hours between groups (19.2 ± 3.3 versus 16.3 ± 4.3 h/week), maternal urea was measurably lower in the pregnancies with successful outcomes (16.2 mmol/L versus 23.9 mmol/L). In addition, maternal urea showed a negative correlation with both birth weight and gestation at delivery with a maternal serum urea < 17.5 mmol/L (48 mg/dL) correlating with delivery after 32 weeks’ gestation and birth weight greater than 1500 g [209]. A graded increase in dialysis guided by residual renal function and biochemical parameters was also reported by Luders et al. Weekly hours of haemodialysis were initially prescribed according urine output (1 L), time on dialysis prior to pregnancy (1 year) and weight (70 kg), then increased according to mid-week pre-dialysis serum urea, blood pressure, weight gain, polyhydramnios and uraemic symptoms. Mean weekly dialysis was 17.6 ± 2.9 h/week. Multivariable linear regression identified a midweek pre-dialysis serum of urea 12.5 mmol/L (BUN 35 mg/dL) as discriminatory in determining successful pregnancy outcome. The use of Kt/V or equivalent renal clearance has not been validated in pregnancy and should not be used as measure of dialysis adequacy in pregnancy [210]. Expert consensus is that clinical assessment of the ultrafiltration target is performed at least weekly, in order to accommodate anticipated weight-gain in pregnancy of 300 g/week during the second trimester and 300–500 g/week in the third trimester [210], with a post-dialysis blood pressure target < 140/90 mmHg, whilst avoiding intradialytic hypotension < 120/70 mmHg [211].

The provision of long, frequent haemodialysis has implications for electrolyte and nutritional provision and pregnant women on dialysis should have access to nutritional assessment and dietary counselling. Nutritional support is considered in many publications on dialysis in pregnancy, although data are diverse with different nutrients reported across the various studies, and no consensus in the vitamins and microelements that should be routinely monitored [210]. Expert consensus is that diet of women receiving long, frequent haemodialysis should be unrestricted and rich in protein (1.5–1.8 g/kg IBW/day [211]). Electrolytes including magnesium and calcium-phosphate balance should be monitored every 1–2 weeks [211]. Dialysate concentrations of potassium, calcium and phosphate may need to be increased. Magnesium supplementation may be required. Dialysate losses of folic acid and water-soluble vitamins need to be considered, with increased supplementation as required, including high-dose folic acid (5 mg) pre-pregnancy (whenever possible) and in the first trimester.

There are inadequate data to confirm the efficacy, safety and equivalence of peritoneal dialysis in supporting pregnancy, compared to enhanced haemodialysis. A systematic review of 38 pregnancies in women receiving peritoneal dialysis prior to pregnancy reported fetal survival in 83% of pregnancies, with 39% delivering prior to 34 weeks and 65% of babies being small for gestational age [206]. Continuing peritoneal dialysis may be considered in the context of vascular access difficulties, logistical barriers to frequent haemodialysis and good residual renal function. Alternatively, a combined approach of peritoneal dialysis supplemented by intermittent haemodialysis during pregnancy has been reported [212, 213].

##### Initiating dialysis during pregnancy

**Guideline 5.2.5**

We suggest haemodialysis should be initiated in pregnancy when the maternal urea concentration is 17-20 mmol/L and the risks of preterm delivery outweigh those of dialysis initiation. Gestation, renal function trajectory, fluid balance, biochemical parameters, blood pressure and uraemic symptoms should be considered in addition to maternal urea concentration (2D).

**Rationale**

The guideline committee acknowledges that there are inadequate data to produce evidence-based recommendations for the initiation of dialysis during pregnancy. However a specific request from the UK renal community was received, for expert, opinion-based practice.

In pregnancy, concern regarding the fetotoxicity of urea is likely to precede maternal indications for dialysis, which are the same as outside of pregnancy: refractory hyperkalaemia, acidosis and/or fluid overload, and uraemic symptoms that impact upon daily living [214]. A recommendation to initiate dialysis when maternal urea concentration is greater that 17 mmol/l is extrapolated from historical, observational data reporting high rates of fetal death in women with this level of renal dysfunction [215, 216], although these data also reflect obstetric and renal practice from over 50 years ago. Contemporary practice is variable: from routine commencement of dialysis at a maternal urea above 17 mmol/L [217], to consideration of dialysis only when the urea is consistently above 20 mmol/L. [66] In addition to maternal serum biochemistry, fetal health (including growth profile and polyhydramnios) and maternal wellbeing (including fluid balance, biochemistry, blood pressure and nutrition) will influence initiation of dialysis in pregnancy. It was the consensus of the guideline committee that in the context of deteriorating renal function, a serum urea above 15 mmol/L should initiate conversations about the risks, benefits and logistics of dialysis initiation in pregnancy, weighed with the risks of preterm delivery before dialysis initiation if the gestation is approaching or more than 34 weeks.

Residual renal function is hypothesised to contribute to improved pregnancy outcomes in women commencing dialysis during pregnancy, compared to women established on dialysis prior to pregnancy [218] and intensification of dialysis in pregnancy has not been shown to confer the same benefit in women initiating dialysis in pregnancy as it does for women established on haemodialysis prior to pregnancy [66]. Meta-analysis data demonstrating improved outcomes with intensification of dialysis do not include women starting dialysis after 20 weeks’ gestation and cannot be generalised [206]. In the absence of evidence for intensive haemodialysis in women newly commencing dialysis in pregnancy, the guideline committee advocates a ‘gentle’ start to new dialysis in pregnancy (for example 2 h, three times per week) with titration according to biochemical parameters and maternal and fetal wellbeing.

#### Lupus nephritis and vasculitis

##### Guideline 5.3.1

We recommend that women with lupus or vasculitis should be advised to wait until their disease is quiescent for at least 6 months before conceiving (1B).

**Rationale**

Data from systematic reviews and a meta-analysis consistently report that having active lupus nephritis is associated with adverse pregnancy outcomes [68, 219]. In addition, prospective studies have recently demonstrated that having quiescent disease is associated with good pregnancy outcomes in the majority of women with lupus nephritis [32, 109, 111]. The 2017 European League Against Rheumatism (EULAR) guideline recommends pre-pregnancy counselling for women with SLE to allow risk stratification and highlights active lupus nephritis, history of lupus nephritis, and the presence of antiphospholipid antibodies as major risk factors in pregnancy [220]. Quiescence is also required to enable the optimisation of medications prior to pregnancy [221]. Currently, induction therapy for acute lupus nephritis involves the use of teratogenic agents, namely cyclophosphamide or mycophenolate mofetil, and mycophenolate mofetil is favoured for maintenance therapy. Women should discontinue these medications three months prior to conception (see section 2) and in most cases need to be established on azathioprine for maintenance [222]. Additionally, women need to know which medications should be established pre-pregnancy (for example, hydroxychloroquine), and ensure that their blood pressure is controlled.

##### Guideline 5.3.2

We recommend that all women with lupus should be advised to take hydroxychloroquine in pregnancy unless it is contraindicated (1C).

**Rationale**

The guideline committee recommends that all patients with lupus should take hydroxychloroquine in pregnancy, endorsing EULAR [7, 220, 223] and British Society of Rheumatology (BSR) [221] guidance. In women with anti Ro antibodies, retrospective case-control data show the use of hydroxychloroquine is associated with a reduction in the risk of congenital heart block (OR = 0.28; 95% CI 0.12–0.63) [224], including in the offspring of women with previously affected infants (OR = 0.23; 95% CI 0.06–0.92) [33]. Hydroxychloroquine is associated with a lower risk of lupus flare [36, 221]. A recent prospective study has demonstrated that hydroxychloroquine is associated with less fetal growth restriction [32].

##### Guideline 5.3.3

We recommend that women with lupus be monitored for disease activity during pregnancy (1D).

**Rationale**

The guideline committee endorses EULAR and BSR guidance [220, 221] that women should be monitored for symptoms and signs of clinical lupus flare. Optimal frequency of monitoring in pregnancy is not adequately addressed in current literature [225]. EULAR recommends that there is an assessment of lupus activity at every visit during pregnancy and that renal function is checked every 4–8 weeks, and in suspected flare [220]. Given the risk of lupus flare in pregnancy and the post-partum period, it is the opinion of the guideline committee that clinical assessment for possible flare including symptoms and urine testing should be performed opportunistically at all health care attendances during pregnancy. Increased surveillance for women with new or worsening clinical manifestations, those with serologically active disease, women with a recent change in treatment, and in any woman in whom there is clinical concern should be undertaken by the MDT, or by a clinician with expertise in managing lupus in pregnancy. Serology can be checked, but clinicians need to be aware that complement levels may rise in pregnancy so a fall within the normal range can herald a flare in pregnancy. There are pregnancy specific modifications for both BILAG2004 and SLEDAI scoring systems, which assess disease activity. Distinguishing a flare of lupus nephritis from preeclampsia can be challenging [222]. The MDT should oversee the care of all women with lupus nephritis during pregnancy due to the complexities of diagnosis, combined with the need for early recognition and timely therapy to maintain maternal and fetal health.

##### Guideline 5.3.4

We recommend that women who are positive for anti-Ro (SSA) or anti-La (SSB) antibodies be referred for fetal echocardiography in the second trimester (1C).

**Rationale**

The guideline committee endorses the EULAR and British Society of Rheumatology (BSR) guidance recommending fetal echocardiography from week 16 for women who are positive for anti-Ro (SSA) or anti-La (SSB) antibodies [220, 221]. However, there is debate as to the frequency of monitoring with suggested protocols ranging in their recommendations from weekly, to monthly, to not repeating if normal at 16–18 weeks [226]. The rationale is that surveillance will pick up early stages of heart block allowing timely intervention, yet the optimum treatment for fetal heart block remains unclear. Observational studies suggest that early changes in cardiac function may be reversible with dexamethasone [227], although there is an absence of evidence that increased immunosuppression is beneficial once complete heart block has developed. Open-label trials of intravenous immunoglobulin (IVIg) have failed to show therapeutic benefit [228, 229].

##### Guideline 5.3.5

We recommend women with antiphospholipid syndrome and a history of a confirmed thromboembolic event or previous adverse obstetric outcome (excluding recurrent early fetal loss) receive low molecular weight heparin in pregnancy and for six weeks postpartum (1B).

**Rationale**

The guideline committee endorses recent expert guidance advising aspirin for all women with antiphospholipid syndrome, including those with recurrent early miscarriage; aspirin and low molecular weight heparin prophylaxis for those with of a history mid/late trimester pregnancy morbidity or loss; and high prophylactic or treatment dose low molecular weight heparin for those with prior thrombotic episodes [10, 230]. A useful appraisal of the relevant evidence and a practical approach to treatment is available [231].

##### Guideline 5.3.6

We recommend that steroids, azathioprine, calcineurin inhibitors, intravenous immunoglobulin and plasma exchange can be used to treat lupus in pregnancy (1C).

**Rationale**

The safety of anti rheumatic drugs in pregnancy is comprehensively reviewed in the 2016 EULAR guideline [7]. Standard maintenance therapy for lupus and women with lupus nephritis who are planning pregnancy would be steroids and azathioprine. Non-fluorinated steroids (for example, prednisolone) are metabolized by the placenta, reducing fetal exposure, although the lowest effective dose should be used to prevent maternal side effects. There are data confirming the efficacy and safety of tacrolimus to maintain remission and treat flares of lupus nephritis in pregnancy [232]. The combination of tacrolimus and steroids is diabetogenic and women taking these drugs in isolation or combination should be screened for gestational diabetes. The role of intravenous immunoglobulin (IVIg) in treating immune cytopaenias is established outside of pregnancy and is safe to use in pregnancy, especially when rituximab is avoided due to the risk of neonatal B-cell depletion (see section 2) [233]. The use of IVIg is also described in case studies where infection risk precludes traditional immunosuppression [234].

#### Diabetic nephropathy

##### Guideline 5.4.1

We recommend that women with diabetic nephropathy have optimisation of blood glucose, blood pressure and proteinuria prior to conception (1C).

##### Guideline 5.4.2

We recommend that women with diabetic nephropathy continue angiotensin converting enzyme inhibitors until conception, with regular pregnancy testing during attempts to conceive (1C).

##### Guideline 5.4.3

We recommend that the schedule of care, surveillance and management of women with diabetic nephropathy should be untaken according to national guidelines for diabetes in pregnancy, in addition to specialist monitoring of renal disease in pregnancy (1D).

**Rationale**

Most women with diabetic nephropathy have successful pregnancy outcomes [235–238]. However, diabetic nephropathy in pregnancy is associated with an increased risk of adverse outcomes including pregnancy loss, congenital malformation, pre-eclampsia, preterm delivery, growth restriction and neonatal unit admission; with glycaemic control at the time of conception and the severity of underlying CKD contributing [235, 239–241]. Reassuringly, a European-wide cohort study including 163 pregnancies in women with type 1 diabetes compared to 630 women with type 1 diabetes who did not undertake a pregnancy, showed that pregnancy was not an independent risk factor for the development of microvascular complications [242].

Pre-pregnancy counselling of women with diabetes is associated with improved glycaemic control prior to pregnancy and a reduction in rates of spontaneous pregnancy loss and congenital malformations [243]. Data from single arm studies (*n* = 8–24) show that reduction of pre-pregnancy proteinuria with angiotensin converting enzyme inhibitors (ACEi) is associated with a reduction in proteinuria in pregnancy [244, 245]. Women with diabetes and proteinuria (*n* = 7) treated with ACEi prior to pregnancy to achieve blood pressure < 135/85 mmHg and albuminuria < 300 mg/day have been shown to have pregnancy outcomes comparable to women with diabetes in the absence of nephropathy [246]. The periconceptual use of ACEi is described in section 3.3.5.

Proteinuria increases in pregnancy in the majority of women with diabetic nephropathy, including progression to the nephrotic range [235]. Low molecular weight heparin may be indicated for the prevention of venous thromboembolism, although the level of proteinuria at which the risk of venous thromboembolism becomes clinically significant is unknown (see section 4.5).

The guideline committee endorses national guidance for the management of diabetes in pregnancy [2].

#### Urinary Tract Infection (UTI)

##### Guideline 5.5.1

We suggest women with reflux nephropathy, congenital anomalies of the kidneys and urinary tract (CAKUT), women with CKD taking immunosuppression, and women with a history of recurrent UTI should be offered antibiotic prophylaxis during pregnancy after a single UTI in pregnancy, including asymptomatic bacteriuria (2D).

##### Guideline 5.5.2

We recommend pre-pregnancy UTI prophylaxis be continued in pregnancy using agents known to be safe (1D).

**Rationale**

Asymptomatic bacteriuria is estimated to occur in 2 to 7% of pregnancies with a risk of progression to acute pyelonephritis if untreated. A meta-analysis of studies to 2015 showed that treatment of asymptomatic bacteriuria reduced the incidence of pyelonephritis in pregnancy from 21 to 5% (RR 0.23, 95% CI 0.13–0.41), with some poor quality evidence that antibiotic use also reduced the incidence of low birth weight babies and preterm delivery [247]. Screening of all pregnant women for asymptomatic bacteriuria is therefore recommended [248]. There are no data describing outcomes for women with CKD and asymptomatic bacteriuria in pregnancy.

The prevalence of UTI following renal transplantation varies from 23 to 75% according to diagnostic criteria, length of follow-up and antibiotic prophylaxis [249]. Registry data show that incidence within the first six months is 17% in women, with a cumulative incidence of 60% by three years post-transplantation [250]. Between 3 and 27% of renal transplant recipients experience recurrent UTI [249, 251, 252]. The reported incidence of UTI in pregnancy in women with renal transplants is variable with cohorts reporting incidences of between 14 and 42% [253, 254]. An increased incidence of UTI in pregnancy is also described in women with reflux nephropathy [255–257] and polycystic kidney disease [258]. There are no published data to guide the management and prophylaxis of urinary tract infection specifically in women with CKD in pregnancy.

In the absence of evidence specifically examining women with CKD, the guideline committee endorses generic guidelines for UTI in pregnancy [248, 259–261]. It was the consensus opinion of the guideline committee that the following women with CKD have an increased risk of complicated and/or recurrent UTI in pregnancy: women with reflux nephropathy, women with congenital anomalies of the kidneys and urinary tract (CAKUT), women with CKD on immunosuppression including women with renal transplants, and women with a history of recurrent UTI prior to pregnancy. In the absence of evidence of harm, antibiotic prophylaxis should be offered to these women following a single confirmed UTI, with or without symptoms, in pregnancy. This decision should be informed by urine culture and antimicrobial sensitivities, and patient preference.

Women who have been commenced on UTI prophylaxis prior to pregnancy should continue prophylaxis in pregnancy with an antimicrobial that is considered safe as their risk of infection is likely increased in pregnancy due to gestational changes to the urinary tract including dilatation of the renal pelvis and ureter, decreased ureteral peristalsis, and reduced bladder tone.

Not all antimicrobials are considered safe in pregnancy. Penicillins, cephalosporins, fosfomycin, trimethoprim (not in first trimester) and nitrofurantoin (not at the end of pregnancy, not in glucose-6-phosphate dehydrogenase deficiency and ineffective if pre-pregnancy eGFR < 45 ml/min/1.73m^2^) can be used.

#### Reflux nephropathy and Congenital Abnormalities of the Kidney and Urinary Tract (CAKUT)

##### Guideline 5.6.1

We recommend women with previous bladder surgery (re-implantation of ureter, bladder reconstruction, all complex paediatric urology) should be discussed during pregnancy with a urologist with expertise in bladder reconstruction to evaluate options for delivery (1D).

**Rationale**

The majority of women with previous urinary tract surgery can have healthy, successful pregnancies without compromise to previous urinary tract reconstruction. No long-term adverse outcomes were identified in a case series of 29 pregnancies in the UK, although urinary tract infections (55%) and upper renal tract obstruction (10%) were common [262]. The anatomy of the lower urinary tract following reconstructive bladder surgery can be variable, and the risk of obstruction caused by the gravid uterus, or damage to bladder and ureters during caesarean section, should be anticipated. Caesarean section is not mandatory but can be performed, and when feasible, should be done with input from a urologist with experience in bladder reconstruction [263].

##### Guideline 5.6.2

We recommend that antenatally detected abnormalities in the fetal kidneys and/or urinary tract should be discussed with fetal medicine and paediatric nephrology specialists to determine appropriate neonatal management (1D).

##### Guideline 5.6.3

We recommend that children with antenatally detected abnormalities in the fetal kidneys and/or urinary tract should have specialist follow up if features of urinary tract infection are identified (1C).

**Rationale**

There are inadequate data to define an evidence-based management strategy for antenatally detected abnormalities of the urinary tract. Expert consensus, based on observational data, is that infants of mothers with urinary tract abnormalities, who had normal urinary tracts on antenatal ultrasound scans do not need further follow up unless features of urinary tract infection are identified in childhood [264]. Neonatal management of antenatally detected abnormalities of the urinary tract will be dependent on the severity of the radiologically identified abnormality and clinical features in the newborn.

The inheritance pattern and penetrance of forms of CAKUT from parent to child is poorly defined. Heterogeneous multifactorial genetic traits are likely but monogenic forms of inheritance have also been described. Cohort studies report between 36 and 67% of children of patients with vesicoureteric reflux demonstrate reflux on a voiding cystourethrogram [265, 266]. However, not all vesicoureteric reflux results in renal parenchymal damage and 80% of mild cases resolve by 5 years [267].

## Lay Summary

Most women with chronic kidney disease (CKD) have successful pregnancies, but kidney disease can affect the health of both pregnant women and their babies. For that reason, experts in renal disease and pregnancy should be available to all women with kidney disease in the UK to offer advice before pregnancy and to help support, monitor and treat women with kidney disease when they are pregnant and after delivery.

Planning pregnancy is important for women with CKD. Women with kidney disease should have access to contraception until they are ready to become pregnant and the progesterone-only pill (‘mini pill’), contraceptive implant and Mirena**®** coil are safe and effective. Women should let their nephrologist know that they would like to consider pregnancy before they stop using contraception. Women of reproductive age with CKD should have an opportunity to discuss the likely risks of a pregnancy for both herself and her baby, with a specialist. Risks include delivering her baby before the due date, her baby being small and needing admission to intensive care, and a worsening of her kidney function in or after pregnancy. A woman’s kidney disease and blood pressure should be treated before she conceives. Medications may need to be adjusted so that they are safe for a developing baby. Many drugs are safe in pregnancy including ciclosporin, tacrolimus, hydroxychloroquine and azathioprine. However mycophenolate is harmful to a developing baby and it is important that mycophenolate is swapped for an alternative medication (usually azathioprine) and a woman’s kidney function to be monitored following this change before she tries to conceive. Blood pressure medications that end in ‘pril’ or ‘sartan’ also need to be stopped, either before pregnancy or as soon as possible in early pregnancy (before 12 weeks).

Women with CKD should have specialist care of kidney disease in pregnancy, in addition to routine antenatal care. This means that they will be seen by doctors (obstetricians) as well as midwives during their pregnancy, and they will have extra scans to check the growth and wellbeing of their baby. Many women with CKD will need treatment for their blood pressure in pregnancy and a number of different blood pressure medications are safe including labetalol, nifedipine, methyldopa. Pregnant women with CKD may need iron (which can be given by injection), and vitamin D. If women were taking erythropoietin (‘Epo’) before conceiving then the dose will need to be increased in pregnancy. Women with CKD, including women with renal transplants, can have a vaginal delivery. They can also breastfeed if they wish and can be given medications that are safe in breastfeeding if they need them.

Compared with women without CKD, women with CKD have a higher risk of a condition called pre-eclampsia. This condition only occurs in pregnancy and goes away after delivery. It causes high blood pressure, protein leak into the urine, headaches and abnormal blood tests. It can also affect the growth of the baby. It can be difficult to know if a woman with CKD has pre-eclampsia if she had high blood pressure and protein in her urine before pregnancy, and specialists should be available to help with diagnosis and treatment. If a woman develops pre-eclampsia, she will need close monitoring and she may need to deliver her baby early. All women with CKD should take low-dose aspirin in pregnancy. This is safe and reduces the risk of pre-eclampsia.

Women with kidney transplants, lupus and diabetes can have successful pregnancies. Timing of pregnancy in these conditions is particularly important in order to make sure kidney function is stable, and that lupus and diabetes are well controlled before pregnancy, in order to have the best chance of a healthy baby. Women who have congenital kidney disease and women who have had bladder surgery should have specialist advice on the best way to deliver their baby. Pregnancy while on dialysis has a very high risk of complications. Although the number of hours of dialysis can be increased in pregnancy, pregnancy outcomes are better for most women on dialysis if they can delay pregnancy until after successful kidney transplantation.

## Appendix 1

### The experience of pregnancy and renal disease

#### Experience 1: Multidisciplinary team

Pregnancy with CKD is significantly different from a normal pregnancy and for me it was critical that I was referred to a specialist care team. Within a week of knowing I was pregnant I was referred. The team at the hospital were fantastic, and it gave me great peace of mind to know that my care was in the hands of a multispecialty team who were aware of my health condition and could advise me accurately.

#### Experience 2: Multidisciplinary team

During the pregnancy I attended regular appointments with specialists in kidney disease in pregnancy. I found that I saw less of my usual renal team than I expected, however it was very clear the communication between all teams was excellent.

#### Experience 3: Multidisciplinary team

Possibly the thing of greatest importance was the immense emotional support that I received from the specialist team caring for me. This, I believe, was the most important aspect for my wellbeing during pregnancy. At any point if I was tense about anything, they would sit me down and listen to my concerns patiently. I remember I was under the care of a specialist obstetrician. In an appointment when my blood pressure was rising, he had to increase my dose of labetalol. I was very tense and I started weeping. He just looked into my eyes and said, “I promise it will be fine.” These words will stay with me in my grave. It touched me very deeply and it was like my tension had disappeared immediately.

#### Experience 4: Fertility

As a lupus and renal patient, I have gone through 6 known miscarriages, 3 failed IVF attempts and 2 successful pregnancies. The first successful pregnancy was after 15 years of marriage, which is a long time, I would imagine, by anyone’s standard.

#### Experience 5: Pre-pregnancy counselling

Women with CKD need significant support to understand if pregnancy is something that they should consider in the first place. I first got diagnosed with CKD in 2009. During a visit to the nephrologist in 2011 I was told that I should not think about pregnancy at all, because it would be fatal for me and the baby. I was not given detailed counselling at that point and it was very heart-breaking. However, when I met an expert in 2013 she presented the pros and cons of having a baby with underlying CKD in a very balanced manner. My major concern was my life expectancy and that of my baby and I was assured that shouldn’t be a concern.

#### Experience 6: Pre-pregnancy counselling

Prior to falling pregnant I attended the pre-pregnancy appointment. At this appointment I met the renal transplant doctor who specialised in pregnant transplant patients, a high-risk obstetrician and a specialist in pregnant women with complex medical conditions. At this appointment, which lasted approximately one hour, my medical history was taken and my medication was reviewed. I was then informed of all the potential risks associated with falling pregnant and I was taken off all the medication that could be harmful to my unborn baby and these medications were changed to medication safe to use in pregnancy. I remember walking out of the appointment feeling more comfortable, and confident that I would be provided with appropriate care during my pregnancy, and even though I may encounter more problems than the average pregnant woman, I would have the support and medical care required. Some of the main pieces of information I remember very clearly are that I would be at higher risk for having a premature baby and I would be high-risk for pre-eclampsia, but I was happily surprised to hear that research suggests there would no effects on the baby secondary to me taking immunosuppressive medication in pregnancy. I never expected to be able to have children with a renal transplant.

#### Experience 7: Antenatal care

Regular check-ups were the most critical part in my entire journey through pregnancy. I believe it is important for every pregnant woman with CKD to have such detailed follow-up. I started with visiting the clinic once every month. As the pregnancy progressed, the check-ups were scheduled twice a month and towards the end, they were weekly. These meetings helped relieve a huge amount of anxiety that I had, especially towards the last few weeks of my pregnancy

#### Experience 8: Antenatal care

I spent approximately 8 weeks in hospital before and after I gave birth to my son. I was seeing one doctor or another at least weekly from the second trimester onwards. I also needed to leave work at about 28 weeks pregnant. At first, I felt guilty about work. However, they were extremely supportive and they were the ones to advise I take long-term sick leave. Once I didn’t have to worry about work, I didn’t mind spending time in hospital because I know it was what I needed to do to keep my baby and me safe and healthy.

#### Experience 9: Pre-eclampsia

When I was about 22 weeks pregnant, at the renal clinic, my blood pressure was high. When the consultant measured it again in his room it was still very high, about 180/120. I could see the panic in his eyes. He immediately took me to the delivery suite where I was informed I needed to be monitored. I was informed during this time that it was hard to differentiate between kidney disease and pre-eclampsia due to the symptoms being the same - high blood pressure and protein in the urine. I was also advised that if the risk remained I may need to give birth. I was informed at this stage the baby would have very little chance of survival and even if she did survive would most likely have developmental problems. I was further advised of the option of termination, as I was still under 24 weeks. This was devastating to me, as I had obviously heard of pre-eclampsia and that I was at risk, but had never contemplated it actually happening or its effect.

#### Experience 10: Peripartum care

My obstetrician had always encouraged and anticipated a natural birth but as both my health and the health of my son worsened towards the end of pregnancy I needed to have a C-section. In the end, I was fine with this decision because I felt so unwell that I didn’t think I would be well enough to endure a long labour. I was very short of breath, my heart rate was high around 120–150 at times and I was very weak. I was anxious about losing blood and being able to lie flat for the C-section but I trusted the opinion of all the doctors involved which was reassuring.

#### Experience 13: Dialysis

Things didn’t improve and my creatinine and urea kept creeping up. At that stage my creatinine was about 270 and urea 18. I was finally informed that I had two options: start dialysis whilst pregnant or give birth preterm, following which I may still need dialysis. After being reassured that dialysis would not affect my baby and in actual fact would keep her safe, I opted for the first option of starting dialysis whilst pregnant.

Following this decision I was immediately fitted with a catheter in my neck, which is something I had been dreading. It was done under local anaesthetic and although I didn’t feel any actual pain, the procedure was still very uncomfortable. It took me quite some time to get used to having two wires sticking out of my neck and until now, I don’t like to touch or feel the wires under my skin or to actually see them sticking out of my skin, so I ensure the nurses keep that area covered at all times.

The first time I had dialysis, I was really worried. Although I had been reassured it was pain free and that I wouldn’t feel a thing, the actual thought of being hooked up to a machine that was filtering my blood scared me. Having seen kidney patients in the past, dialysis had always looked ominous to me. However, my first experience was good. I think I was in a good place for anybody to start dialysis for the first time. The nurses there were very sympathetic and experienced and I was amazed by the amount of emphasis on hygiene. I was always attached and taken off the machine by a sister, who was always happy to answer any questions I had. It has also given me a greater respect for nurses, as the ones I encountered were very pleasant whilst at the same time having immense knowledge regarding the whole dialysis process, the machines, and what patients should be doing. I was also amazed at the dialysis machine. How it was able to filter the blood, remove the toxins and then pump the blood back into my body. It also made me realise what an important job the kidneys do.

I soon got used to the process, which was 2 h 3 times a week, then 2.5 h 4 times a week. The effect of the dialysis however was very tiring and although sometimes I fell asleep during it, I still needed a good solid 2 h sleep immediately afterwards. I would still feel exhausted for the remainder of the day with my body feeling very weak. I would then feel much better and rejuvenated the following day before being subjected to dialysis the day after. So it was up and down, feeling tired then very well, then very tired again. This made me wonder how people on dialysis manage to lead a normal life, which I was obviously not doing at that time.

My blood was being taken every time I had dialysis before and after, and I could see for myself the drastic changes it made. After the first session my creatinine went down to about 140 and my urea to about 9. When I heard this I nearly cried. After all the bad news and bad results I had been hearing, to finally hear something positive and that my baby was safe meant so much.

Although I’m now used to the process and keep myself entertained during those hours by reading, working, talking, surfing the net, it remains very hard. The dialysis days do not count as part of my normal life as I still feel quite tired, depending on how much fluid is taken on those days. I still need to go home to 2 children, however, so cannot properly rest. I’m too tired to do anything practical as a mum, such as cook, clean or wash.

## Appendix 2

**Table 3:** Summary of clinical responsibility for elements of the guideline

Recommendations	Primary care and community	Secondary care	MDT
Structure of care			✓
Medication in pregnancy and lactation	✓	✓	✓
Contraception	✓	✓	✓
Fertility	✓	✓	✓
Pre-pregnancy counselling		✓	✓
Optimisation for pregnancy	✓	✓	✓
Assessment of renal function in pregnancy	✓	✓	✓
Antenatal care	✓	✓	✓
Pre-eclampsia prophylaxis	✓	✓	✓
Blood pressure management	✓	✓	✓
Venous thromboembolism	✓	✓	✓
Anaemia	✓	✓	✓
Bone health		✓	✓
Renal biopsy			✓
Peripartum care		✓	✓
Postnatal care	✓	✓	✓
Transplantation		✓	✓
Dialysis			✓
Lupus nephritis and vasculitis		✓	✓
Diabetic nephropathy	✓	✓	✓
Urinary tract infection	✓	✓	✓
Reflux nephropathy and CAKUT		✓	✓

## Appendix 3

### Ovid Medline search terms (1946 to 2018)

**Pregnancy**:

1. exp. Pregnancy/
2. exp. Pregnancy Complications/
3. exp. Pregnancy Trimesters/
4. exp. Pregnancy Outcome/
5. exp. Pregnancy Maintenance/
6. exp. Pregnancy, Multiple/
7. exp. Pregnancy, High-Risk/
8. exp. Pregnant Women/
9. exp. Delivery, Obstetric/
10. exp. Postpartum Period/
11. exp. Peripartum Period/
12. exp. Gravidity/
13. pregnan$.mp.
14. gravid.mp.
15. gestation$.mp.
16. c?esarean$.mp.
17. obstetric.mp.
18. peripartum.mp.
19. intrapartum.mp.
20. postpartum.mp.
21. (pregnan$ or gestation$ or pre-eclamp$ or preeclamp$ or pre eclamp$ or eclamp$ or HELLP or obstetric$ or postpartum$ or peripartum$ or intrapartum$ or antepartum$ or prepartum$ or antenatal$ or prenatal$ or postnatal$ or perinatal$ or internatal$ or cesarean$ or caesarean$).ti.
22. or/1–21
23. limit 22 to (English language and female)

**CKD:**

1. exp. Renal Insufficiency/
2. exp. Kidney Diseases/
3. exp. Kidney Failure, Chronic/
4. ((Kidney* or renal*) adj4 (disease* or failur* or transplant* or insufficienc* or implant*)).tw.
5. CKD.tw.
6. kidney diseases/ and (chronic or end-stage or endstage).ti,ab.
7. renal insufficiency/ and (chronic or end-stage or endstage).ti,ab.
8. ((chronic or progressive) adj2 (renal or kidney)).ti,ab.
9. (chronic adj (kidney or renal) adj insufficienc*).ti,ab.
10. ckd.ti,ab.
11. ((renal adj3 insufficienc*) not (acute adj2 renal)).ti,ab.
12. ((renal or kidney) and chronic).ti,ab.
13. or/1–14

**Transplant:**

1. exp. Kidney Transplantation/
2. ((kidney? or renal$) adj3 (transplant$ or graft$)).ti,ab.
3. or/1–2

**Lupus and GN:**

1. exp. glomerulonephritis/
2. exp. nephritis/
3. Glomerulonephritis.mp.
4. (glomerul$ adj3 nephritis).mp.
5. gn.mp.
6. (iga or berger$ or (focal adj3 glomerul$) or (segmental adj3 glomerul$) or fsgs or minimal change or (membran$ and glomerul$) or mgn or mcgn or dense deposit disease or mesangial proliferative glomerul$ or alport$ or hereditary nephr$ or (rapid$ progressive adj3 glomerul$) or crescentic gn).mp.
7. glomeruloneph$.ti,ab.
8. nephropath$.ti,ab.
9. (glomerul$ adj (sclerosis or nephritis)).ti,ab.
10. exp. lupus nephritis/
11. lupus nephritis.mp.
12. or/1–9.

**Dialysis** (Iansavichus et al., 2015, #75528)**:**

1. ((dialy$ OR h?emodia$).mp.
2. ((end stage OR endstage) adj (kidney OR renal)).tw.
3. esrd.tw.
4. renal replacement.mp.
5. ur?emi$.mp.
6. ur?emi$.tw.
7. exp. *Uremia/
8. capd.tw.
9. h?emofilt$.mp.
10. ur?emic patient$.tw.
11. intradialy$.tw.
12. tenckhoff$.tw.
13. ccpd.tw.
14. ur?emic.tw.
15. fistula$.mp
16. or/1–15

**Contraception:**

1. exp. contraception/
2. exp. contraception behavior/
3. exp. contraceptive devices/
4. contracept$.mp
5. exp. family planning/
6. family planning.mp
7. exp. pregnancy, unplanned/
8. exp. pregnancy, unwanted/
9. exp. birth control/
10. birth control.mp.
11. (family planning or family-planning).mp.
12. contracept$.mp.
13. (sexual and reproductive health information).mp.
14. (family planning or planned parenthood or birth control or reproductive health).mp.
15. (birth regulat* or population regulat* or fertility regulat* or birth spacing or pregnancy inter*).mp.
16. fertility control.mp.
17. reproduct$ control.mp.
18. ((unplan* or unwant* or mistime* or wanted* or unintend* or intend*) adj pregnan*).mp.
19. safe sex.tw.
20. ((birth control or family planning or reproductive health) adj clinic).mp.
21. pregnan$ adj2 (prevent* or interrupt* or terminat*).mp.
22. exp. contraception, barrier/
23. exp. condoms/
24. (barrier method* or condom* or vaginal sponge* or cervical cap*).mp.
25. exp. contraception, immunologic/
26. exp. reproductive-control-agents
27. ovulat* adj2 (supress* or inhibit* or prevent*)
28. Birth control pill.mp.
29. Oral contracept$.mp.
30. (intrauterine device* or intra-uterine device* or IUD* or TCu380a or CuT-200 or gynefix or mirena or intrauterine system).mp.
31. exp. contraception, postcoital/
32. morning after pill.mp.
33. emergency contracept$.mp.
34. ((contra* or family planning or pill or method) adj (method or failure or method failure or discontinuation)).mp.
35. ((female or woman or women) adj sterili*).mp.
36. periodic* abstinen* or sexual* abstinen* or coitus interruptus
37. or/1–36

**Fertility:**

1. (fertil* or steril* or infertile* or sub-fertil* or fecund* or subfecund* or sub-fecund* or assist* reproduce*).tw.
2. exp. infertility/
3. Infertility, Female/
4. Anovulation/
5. anovulat*.tw.
6. (oligo-ovulation or “oligo ovulation” or oligoovulat*).tw.
7. exp. fertility/
8. fertil$.ti,ab,sh,tw.
9. infertil$.ti,ab,sh,tw.
10. subfertil$.ti,ab,sh,tw.
11. exp. ovulation induction/ or exp. superovulation/
12. (ovulat$ adj2 induc$).tw.
13. (ovar$ adj2 stimulat$).tw.
14. superovulat$.tw.
15. or/1–14

**Fluid Balance:**

1. Fluid Therapy/
2. Fluid?.ti,ab.
3. Infusions, Intravenous/
4. ((intravenous$ or IV or drip?) adj3 infusion?).ab,ti.
5. Rehydration Solutions/
6. (re-hydrat$ or rehydrat$).ab,ti.
7. (type? adj3 (intravenous$ or IV or drip? or fluid?)).ab,ti.
8. ((rate? or amount? or volume?) adj3 (intravenous$ or IV or drip? or fluid? or admin$)).ab,ti.
9. Body Water/
10. Water-Electrolyte Balance/
11. Water-Electrolyte Imbalance/
12. ((body or bodies) adj2 water).ti,ab.
13. ((fluid? or water$ or electrolyte) adj3 (balanc$ or imbalanc$)).ti,ab.
14. Furosemide/
15. Dopamine/
16. Dopamine Agents/
17. Furosemide.mp.
18. Dopamine.mp.
19. or/1–18

**Anaemia:**

1. exp. anemia/
2. (anemi* or anaemi*).ti,ab.
3. *Anemia/ OR an?emi$.ti.
4. erythropoietin$.mp.
5. or/1–4

**Bone:**

1. exp. hyperparathyroidism, secondary/
2. ((renal adj2 osteo*) or ((renal or secondary) adj2 hyperparathyroidism)).ti,ab.
3. hyperparathyroidism.mp.
4. or/1–3

**Diabetic nephropathy:**

1. exp. pregnancy in diabetics/
2. pregnancy in daibetics.mp.
3. (diabetic adj (kidney or renal) adj (disease* or failure)).ti,ab.
4. exp. diabetic nephropathies/
5. diabetic nephropathy.mp.
6. or/1–6

**Hypertension:**

1. Chronic hypertensi*/or chronic hypertension in pregnanc*
2. exp. Hypertension/
3. exp. hypertension, renal/
4. exp. Blood Pressure/
5. exp. Blood Pressure Determination/
6. exp. Antihypertensive Agents/
7. Hypertens*.mp.
8. pre-eclamp*.mp.
9. preeclamp*.mp.
10. toxemia*.mp.
11. toxaemia*.mp.
12. gestosis.mp.
13. antihypertensive*.mp.
14. ((high$ or rais$ or elevat$ or heighten$ or increas$) adj3 (blood pressure or diastolic pressure or systolic pressure or pulse pressure)).mp.
15. ((high$ or rais$ or elevat$ or heighten$ or increas$) adj3 (BP or DBP or SBP)).mp.
16. or/1–15

**Urinary Tract Infection and Reflux:**

1. exp. urinary tract/
2. exp. urinary tract infections/
3. exp. cystitis/
4. vesico-ureteral reflux/
5. exp. pyelonephritis/
6. exp. Urinary Calculi/
7. exp. Vesico-Ureteral Reflux/
8. exp. Urogenital Abnormalities/
9. exp. Urethritis/
10. (UTI or CAUTI or RUTI or cystitis* or bacteriuria* or pyelonephriti* or pyonephrosi* or pyelocystiti* or pyuri* or VUR or urosepsis* or uroseptic* or urosepses* or urethritis*).ti,ab.
11. ((urin* or renal* or kidney*) adj1 (system* or tract* or calculus or calculi* or stone* or sepsis*)).ti,ab.
12. ((bladder* or genitourin* or genito urin* or kidney* or pyelo* or renal* or ureter* or ureth* or urin* or urolog* or urogen*) adj3 (infect* or bacteria* or microbial* or block* or obstruct* or catheter* or inflamm*)).ti,ab.
13. ((upper or lower) adj3 urin*).ti,ab.
14. ((vesicorenal* or vesicoureteral* or vesicoureteric* or vesico renal* or vesico ureteral* or vesicoureteric* or bladder* or cystoureteral* or ureter* or urether* or nephropathy*) adj3 (backflow* or reflux*)).ti,ab.
15. CAKUT.ti,ab.
16. or/1–12

**Venous Thromboembolism:**

1. pulmonary embolism/ or thromboembolism/ or venous thromboembolism/ or venous thrombosis/ or upper extremity deep vein thrombosis/
2. (((venous or vein) adj (thrombosis or thromboses or thrombus or thromboembolism)) or (dvt or vte) or ((pulmonary or lung) adj3 (embolism or emboli or embolus or emboliz* or thromboembolism))).ti,ab.
3. or/1–2

**Proteinuria:**

1. exp. Proteinuria/
2. exp. Albuminuria/
3. protein creatinine ratio.mp.
4. protein to creatinine ratio.mp.
5. protein: creatinine ratio.mp.
6. uPCR.mp.
7. spot urinary protein creatinine ratio.mp.
8. albumin creatinine ratio.mp.
9. albumin to creatinine ratio.mp.
10. albumin: creatinine ratio.mp.
11. uACR.mp.
12. spot urinary albumin creatinine ratio.mp.
13. microalbuminuria.mp.
14. exp. Nephrotic Syndrome/
15. or/1–14

**Biopsy:**

1. exp. Biopsy, Fine-Needle/
2. exp. Biopsy/
3. exp. Biopsy, Needle/
4. exp. Biopsy, Large-Core Needle/
5. morphology.mp.
6. exp. Pathology/
7. exp. Pathology, Molecular/
8. exp. Pathology, Clinical/
9. exp. Histology/
10. or/1–9

**Renal Function:**

1. exp. creatinine/
2. exp. kidney function tests/
3. exp. glomerular filtration rate/
4. kidney function.mp.
5. renal function.mp.
6. glomerular filtration.mp.
7. GFR.mp.
8. (CKDEPI or CKD-EPI or Chronic Kidney Disease Epidemiology).mp.
9. (MDRD or modification of diet in renal disease).mp.
10. Cockcroft.mp.
11. exp. cystatin C/
12. ((equation or formula) AND (kidney function or renal function)).mp
13. or/1–12

**Pre-pregnancy:**

1. exp. Prenatal Care/
2. exp. Preconception Care/
3. pre-natal.ti,ab.
4. pre-concept*.ti,ab.
5. peri-concept*.ti,ab.
6. pre-gestation*.ti,ab.
7. pregestation*.ti,ab.
8. (pre-pregnancy or prepregnancy or prenatal or pre-natal or preconcept* or pre-concept* or periconcept* or peri-concept* or pre-gestation* or pregestation*).ti,ab.
9. ((Counsel* or advice* or education) adj3 (pre-pregnancy or prepregnancy or prenatal or pre-natal or preconcept* or pre-concept* or periconcept* or peri-concept* or pre-gestation* or pregestation*)).mp.
10. (pre-pregnancy or prepregnancy or prenatal or pre-natal or preconcept* or pre-concept* periconcept* or peri-concept* or pre-gestation* or pregestation*) AND (service* OR counsel* OR program* OR care OR education* OR clinic*).mp.
11. or/1–10

**Antenatal:**

1. exp. Perinatal Care/
2. exp. Obstetrics/
3. exp. Maternal Health Services/
4. exp. Home Childbirth/
5. ((midwif* or nurs* or obstetric* or medical*) adj (service* or care)).mp.
6. ((antenatal* or prenatal* or matern* or perinatal* or pregnan* or childbirth* or childbearing*) adj (service* or care)).mp.
7. exp. Midwifery/
8. exp. Nurse Midwives/
9. exp. Birthing Centers/
10. ((nurs* or midwif* or obsteric* or medical*) adj model*).mp.
11. ((nurs* or midwif* or obsteric* or medical*) adj2 team*).mp.
12. (multidisciplinary adj team*).mp.
13. (shar* adj2 care).mp.
14. ((nurs* or midwif* or obsteric* or medical*) adj2 manag*).mp.
15. ((nurs* or midwif* or obsteric* or medical*) adj2 led*).mp.
16. or/1–15

**Postnatal:**

1. exp. Postnatal Care/
2. exp. Postpartum Period/
3. exp. Peripartum Period/
4. postnatal.mp.
5. postpartum.mp.
6. peripartum.mp.
7. post adj2 pregnan*.mp.
8. or/1–8

**Medication:**

Generic and brand drug names (UK, EU and US) in conjunction with drug class were searched as multipurpose fields in conjunction with pregnancy search terms.
